# Two assembly modes for SIN3 histone deacetylase complexes

**DOI:** 10.1038/s41421-023-00539-x

**Published:** 2023-04-19

**Authors:** Chengcheng Wang, Zhouyan Guo, Chen Chu, Yichen Lu, Xiaofeng Zhang, Xiechao Zhan

**Affiliations:** 1 Westlake Laboratory of Life Sciences and Biomedicine, Hangzhou, Zhejiang, China; 2 Key Laboratory of Structural Biology of Zhejiang Province, School of Life Sciences, Westlake University, Hangzhou, Zhejiang, China; 3 Institute of Biology, Westlake Institute for Advanced Study, Westlake University, Hangzhou, Zhejiang, China

**Keywords:** Cryoelectron microscopy, Histone post-translational modifications

## Abstract

The switch-independent 3 (SIN3)/histone deacetylase (HDAC) complexes play essential roles in regulating chromatin accessibility and gene expression. There are two major types of SIN3/HDAC complexes (named SIN3L and SIN3S) targeting different chromatin regions. Here we present the cryo-electron microscopy structures of the SIN3L and SIN3S complexes from *Schizosaccharomyces pombe* (*S. pombe*), revealing two distinct assembly modes. In the structure of SIN3L, each Sin3 isoform (Pst1 and Pst3) interacts with one histone deacetylase Clr6, and one WD40-containing protein Prw1, forming two lobes. These two lobes are bridged by two vertical coiled-coil domains from Sds3/Dep1 and Rxt2/Png2, respectively. In the structure of SIN3S, there is only one lobe organized by another Sin3 isoform Pst2; each of the Cph1 and Cph2 binds to an Eaf3 molecule, providing two modules for histone recognition and binding. Notably, the Pst1 Lobe in SIN3L and the Pst2 Lobe in SIN3S adopt similar conformation with their deacetylase active sites exposed to the space; however, the Pst3 Lobe in SIN3L is in a compact state with its active center buried inside and blocked. Our work reveals two classical organization mechanisms for the SIN3/HDAC complexes to achieve specific targeting and provides a framework for studying the histone deacetylase complexes.

## Introduction

The switch-independent 3 (SIN3)/histone deacetylase (HDAC) complexes are chromatin modifiers that catalyze local histone deacetylation and regulate global gene expression. The SIN3/HDAC complexes, expressed in all eukaryotes, are associated with numerous cellular processes, including embryonic development, cell cycle, cell proliferation, and senescence, or in diseases such as cancer^1–6^.

The highly conserved, multidomain-containing protein Sin3 is thought to provide a platform for the assembly of HDACs and non-catalytic subunits, forming two major types of the SIN3/HDAC complexes (SIN3L and SIN3S)^7–10^. The SIN3L complex, consisting of 10–15 protein components with a combined molecular weight of ~1 megadaltons (MDa), is broadly recruited to the promoter of target genes through DNA-binding factors or other corepressors to inhibit transcription^11–14^. Moreover, the ~0.6 MDa SIN3S complex contains only five known proteins and targets the transcribed regions to suppress intragenic transcription initiation^15–17^.

Since the discovery of the SIN3L and the SIN3S complexes we have gained considerable insights into their functional analysis based on extensive genetic and biochemical studies^9,18–28^. Recently, dominant loss-of-function mutations in human SIN3 proteins were identified as one of the genetic causes responsible for the intellectual disability and craniofacial dysmorphism characteristic of the rare neurodevelopmental disorder Witteveen-Kolk syndrome (WITKOS)^29–31^. In addition, the human SIN3/HDAC complex regulates the expression of several genes important in breast cancer and estrogen signaling, revealing a potential therapeutic strategy^28,32,33^. However, structural information for SIN3/HDAC complex has been slow to emerge, severely limited to only several crystallographic or NMR structures of the isolated domains^34–38^. It remains unclear how the other subunits collaborate with Sin3 to furnish the complex organization, as well as how the HDAC associates with Sin3 to enable the deacetylase activity. Addressing these questions necessitates the entire structure of SIN3/HDAC complex.

Here we report the atomic cryo-electron microscopy (cryo-EM) structures of the SIN3L and SIN3S complexes from *Schizosaccharomyces pombe* (*S. pombe)*. The structure information reveals two distinct representative scaffolds of the SIN3/HDAC complex, and allows the mechanistic understanding of the molecular basis for the underlying SIN3L/SIN3S function.

## Results

### Structure determination of SIN3L and SIN3S

**Fig. 1:**
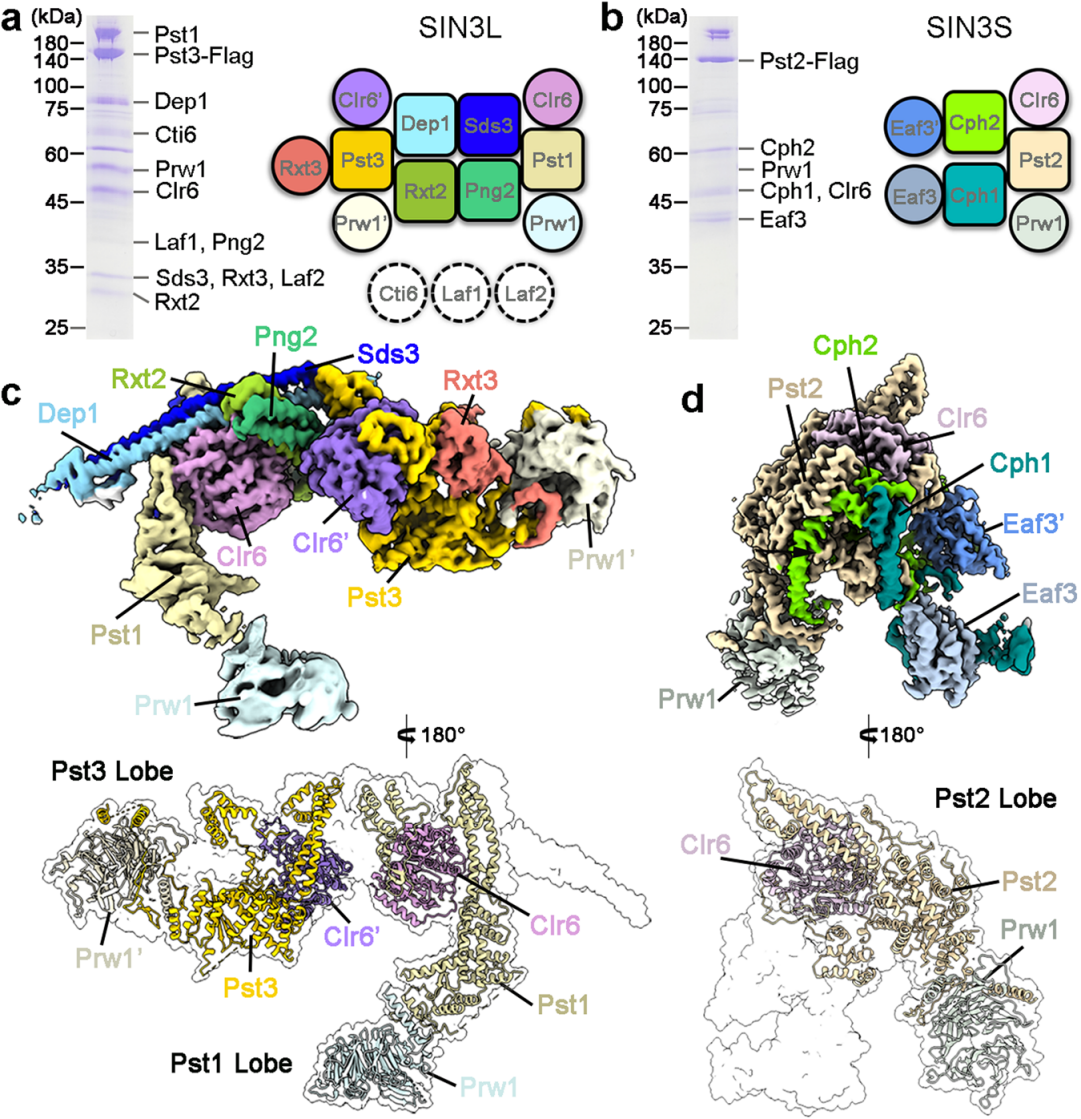
Structure determination of the SIN3L and SIN3S complexes from *S. pombe*. **a** Purification and schematic view of the SIN3L complex. The components of SIN3L were separated on 12% SDS-PAGE and further confirmed by mass spectrometry analysis. The schematic diagram shows the subunit organization of the SIN3L complex. Three components (Cti6, Laf1, and Laf2), present in a purified sample, are labeled in dotted circles for not included in the final model. **b** Purification and schematic of the SIN3S complex. The components of SIN3S were separated on 12% SDS-PAGE and further confirmed by mass spectrometry analysis. The schematic diagram shows the subunit organization of the SIN3S complex. **c** Overall structure of the SIN3L complex. The 3.2-Å EM map is shown in the upper panel with individual component color-coded identically. In the lower panel, the Pst1 Lobe (containing Pst1, Clr6, and Prw1) and the Pst3 Lobe (containing Pst3, Clr6’, and Prw1’) are present as a colored cartoon with the whole complex in transparent surface. **d** Overall structure of the SIN3S complex. The 2.9-Å EM map is shown in the upper panel with individual component color-coded identically. In the lower panel, the Pst2 Lobe (containing Pst2, Clr6, and Prw1) is present as a colored cartoon with the whole complex in transparent surface. All structural images in this paper were generated in ChimeraX^64^ and PyMOL^65^.

We sought to purify the endogenous SIN3/HDAC complexes from *S. pombe* by employing a C-terminal Flag-tag on the Sin3 homolog Pst1, Pst2, or Pst3. Cryo-EM analysis finally yielded a reconstruction of SIN3L at an average resolution of 3.2 Å from the Pst3-Flag yeast strain and a map of the SIN3S at 2.9 Å resolution from the Pst2-Flag yeast strain (Fig. 1a, b; Supplementary Figs. S1–S3 and Table S1).

The high-quality EM density maps allowed protein assignment and de novo atomic modeling, covering 11 molecules for the SIN3L complex and seven molecules for the SIN3S complex. In the structure of SIN3L, Pst1, and Pst3 combined with two copies of the histone deacetylase Clr6 (Clr6 and Clr6’), as well as two copies of the WD40 domain-containing protein Prw1 (Prw1 and Prw1’), and also Dep1, Sds3, Rxt2, Png2, and Rxt3 to form an intricate and asymmetric ‘Large’ scaffold (Fig. 1c; Supplementary Fig. S4). The three other subunits (including Cti6, Laf1, and Laf2) were present in the purified sample, but could not be precisely assigned in the final model, likely due to their intrinsic flexibility. In the structure of SIN3S, Pst2 organizes Clr6, Prw1, Cph1, Cph2, and two copies of Eaf3 (Eaf3 and Eaf3’), assembling into a delicate ‘Small’ scaffold (Fig. 1d; Supplementary Fig. S5).

Notably, the three isoforms of Sin3 (Pst1, Pst2, and Pst3) respectively create a lobe (Pst1/Pst3 Lobes in SIN3L, and a Pst2 Lobe in SIN3S) by directly interacting with Clr6 and Prw1. In addition, other subunits of SIN3L and SIN3S play pivotal roles in maintaining structural integrity. This will be discussed in detail in the following sections.

### Organization of SIN3L

**Fig. 2:**
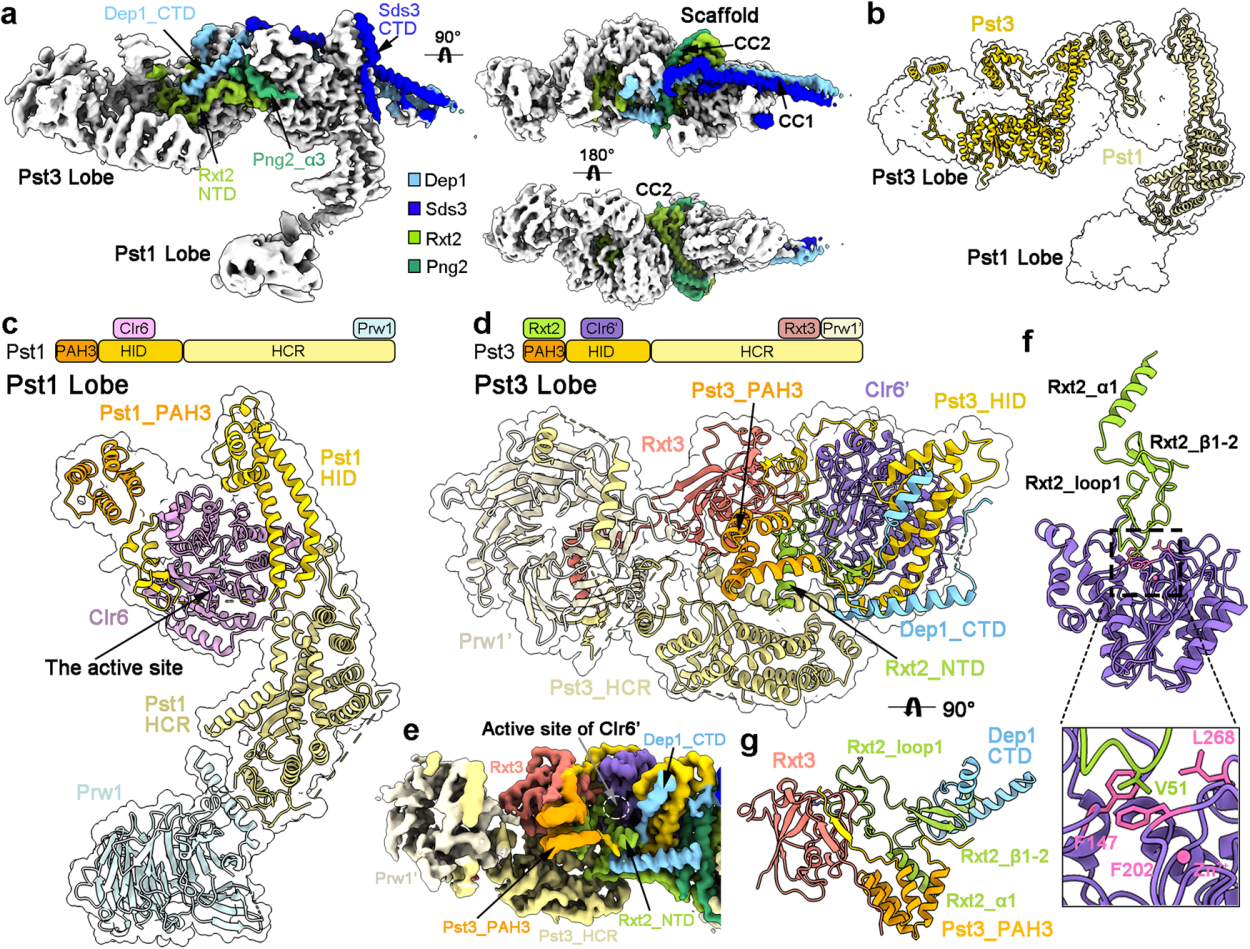
Organization of the SIN3L complex. **a** The scaffold of the SIN3L complex. The scaffold proteins (including Sds3, Dep1, Rxt2, and Png2) are shown in colors with the Pst1/3 Lobe shown in gray. **b** Structures of Pst1 and Pst3 in the two lobes. **c** Structural features of the Pst1 Lobe. A cartoon model of the Pst1 Lobe in an extended conformation is shown in the upper panel. The Pst1_HID stabilizes the Clr6, and the Pst1_HCR interacts with Prw1. The active site of Clr6 is exposed to the exterior space. **d** Structural features of the Pst3 Lobe. A cartoon model of the Pst3 Lobe in a compact state is shown in the upper panel. The Pst3_HID stabilizes the Clr6’, and the Pst3_HCR interacts with Prw1’. The Pst3_PAH3 binds to the Rxt2_α1. The active site of Clr6’ is buried inside and blocked by the Rxt2_NTD. **e** The EM map of the Pst3 Lobe with protein components color-coded. **f** The close-up views of the interactions between Clr6’ and Rxt2_NTD. **g** The Rxt2_NTD is further stabilized in the Pst3 Lobe.

The SIN3L complex adopts an asymmetric architecture, containing three modules: the Pst3 Lobe (Pst3, Clr6’, Prw1’, and Rxt3), the Pst1 Lobe (Pst1, Clr3, and Prw1), and a scaffold (Sds3, Dep1, Rxt2, and Png2) (Fig. 2a). The scaffold, formed by two coiled-coil domains from the Sds3/Dep1 (CC1) and the Rxt2/Png2 (CC2), bridges the Pst1 Lobe and the Pst3 Lobe (Fig. 2a; Supplementary Fig. S6). In addition, the C-terminal domain (CTD, containing α3–4) of Dep1 and the N-terminal domain (NTD, containing α1, loop1, and β1–2) of Rxt2 stretch into the Pst3 Lobe; while the Png2_α3 and the Sds3_α3–4 cover the surface of the Pst1 Lobe. These structural features are consistent with the previous reports that depletion or mutations of these proteins (Sds3, Dep1, Rxt2, and Png2) impair the integrity and function of the complex^39–42^.

Sin3 protein consists of three paired amphipathic α-helices domains (PAH1/2/3), an HDAC interaction domain (HID), and a highly conserved C-terminal region (HCR)^8,43^. In the structure of SIN3L, the segment spanning the PAH3, HID, and HCR of Pst1 or Pst3 is well resolved and reveals a different conformation in the organization of the Pst1 Lobe or the Pst3 Lobe (Fig. 2b–d). The HID of Pst1 or Pst3 stabilizes Clr6 in each lobe in a similar way, which will be discussed in detail in a subsequent section. Superposed by the HIDs, both the PAH3s and the HCRs undergo a rotation of over 90 degrees (Supplementary Fig. S7a). In addition, the hydrophobic pocket of the Pst3_PAH3 associates with Rxt2_α1 (Fig. 2d, e), while that of Pst1_PAH3 is occupied by its own N-terminal loop (Fig. 2c; Supplementary Fig. S7b). Whereas the Pst1_HCR is extended to bind to the Prw1 (Fig. 2c), the Pst3_HCR rotates to interact with the Rxt3, together stabilizing the Prw1’ (Fig. 2d; Supplementary Fig. S7c).

### The deacetylase center of the Pst3 Lobe in SIN3L is blocked

The Pst1 Lobe presents an extended appearance with the active site of Clr6 exposed to the exterior space (Fig. 2c); however, the Pst3 Lobe is in a compact state with the active site of Clr6’ buried inside and blocked by the Rxt2_NTD (Fig. 2d–f). The Rxt2_NTD is unambiguously resolved in the EM density map (Supplementary Fig. S4) and stabilized by the following structural features: the α1 and β1–2 of Rxt2 are anchored in the Pst3 Lobe by the Pst3_PAH3 or the Dep1_α3, respectively; the Rxt2_loop1 is further stabilized by the Rxt3 (Fig. 2g; Supplementary Figs. S7b, S8a–c). Notably, the Rxt2_loop1 is inserted into the pocket of the active site with the residue Val51 stacked against the residues Phe147, Phe202, and Leu268 of Clr6 (Fig. 2f, inset). Moreover, compared with the structures of HDAC bound to the classical inhibitors (TSA or SAHA), the residues 51–54 of Rxt2 occupy the binding site of the inhibitor molecules^44^ (Supplementary Fig. S8d), indicating that the deacetylase activity of Clr6’ in the Pst3 Lobe is blocked.

### Organization of SIN3S

**Fig. 3:**
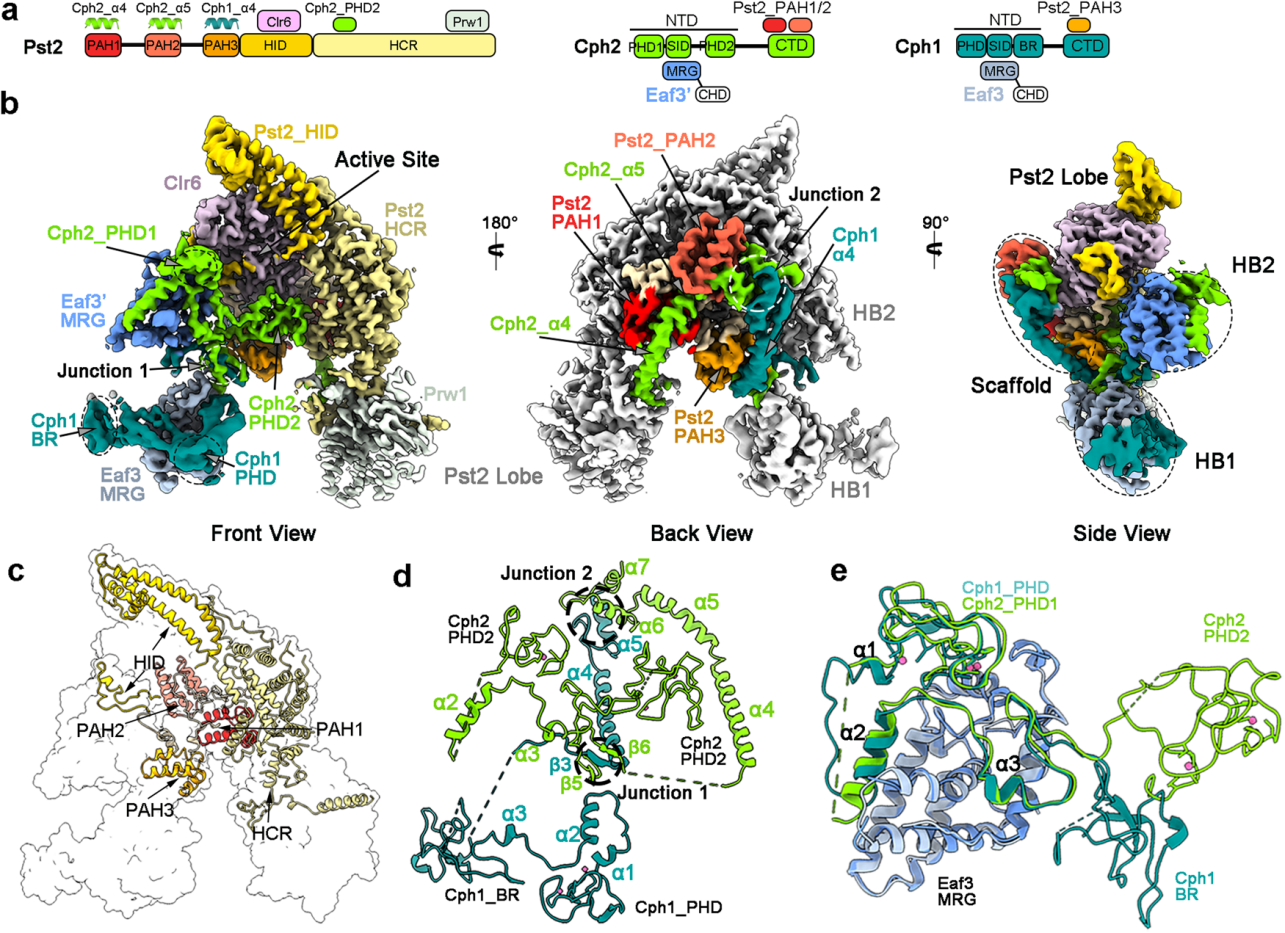
Organization of the SIN3S complex. **a** The domain organizations of Pst2, Cph2, and Cph1. **b** The EM map of the SIN3S complex is shown in three different views. The SIN3S complex contains four modules: Pst2 Lobe (Pst2_HID/HCR, Clr6, and Prw1), two histone binding modules (HB1, consisting of Cph1_NTD and Eaf3; and HB2, consisting of Cph2_NTD and Eaf3’), and scaffold (Pst2_PAH1-3, Cph1_CTD, and Cph2_CTD). **c** The structure of Pst2 (shown in a cartoon model) in the SIN3S complex (shown in the surface model). **d** The structure of Cph1 and Cph2. The CTDs of Cph1 and Cph2 form the scaffold with the PAH1/2/3 of Pst2. The NTDs of Cph1 and Cph2 recruit the MRG domains of Eaf3 and Eaf3’ through their SID domains (α2–3), forming the HB1 and HB2 modules, respectively. **e** The structures of HB1 and HB2 are superposed by the MRG domains. Cph1_PHD is aligned well with the Cph2_PHD1, while the Cph2_PHD2 is away from the BR domain of Cph1.

Unlike the SIN3L complex, the SIN3S complex reveals a compact organization with four modules: the Pst2 Lobe (Pst2_HID/HCR, Clr6, and Prw1), two histone binding modules (the HB1 module, consisting of Cph1_NTD and Eaf3, and the HB2 module, consisting of Cph2_NTD and Eaf3’), and a scaffold (Pst2_PAH1–3, Cph1_CTD, and Cph2_CTD) (Fig. 3a, b). Cph1, Cph2, and the full length Pst2 play essential roles in stabilizing the overall conformation of the SIN3S complex (Fig. 3b; Supplementary Figs. S9, S10a), which is consistent with the previous biochemical and genetic results^23,24^.

In the structure of the SIN3S, all five domains (PAH1/2/3, HID, and HCR) of Pst2 are well resolved and could be modeled (Fig. 3c; Supplementary Fig. S9). Similar to the Pst1 in the SIN3L, the HID of Pst2 binds to the Clr6 and the HCR interacts with Prw1, forming the Pst2 Lobe. The PAH1/2/3 of Pst2 respectively stabilizes Cph2_α4, Cph2_α5, or Cph1_α4 in the rear side of the deacetylase activity center, constructing the scaffold to bridge the Pst2 Lobe and the HB1/2 modules (Fig. 3b, Side View; Supplementary Fig. S10a, b). In addition, the Cph1_β3 forms a β-sheet with the Cph2_β5–6, and the Cph1_α5 interacts with the Cph2_α6–7, forming two junctions to further stabilize the whole complex (Fig. 3b, d).

### The SIN3S contains two histone-binding modules

Cph1 and Cph2 in the SIN3S from *S. pombe* are homologs of the *S. cerevisiae* Rco1 and the human PF1, and contain PHD (plant homeodomain) zinc fingers in their N-terminus^23^, which was proven to serve as the sequence-specific histone recognition element^45^. Cph2 has two PHDs (PHD1 and PHD2), but Cph1 has one PHD and another basic region (BR) with many positive-charged residues corresponding to the Cph2_PHD2 (Fig. 3a, e; Supplementary Fig. S11a).

The Cph1_NTD (consisting of PHD, SID, and BR) and the Cph2_NTD (consisting of PHD1, SID, and PHD2) respectively recruit the MRG domains of Eaf3 and Eaf3’ through their SIDs (α2–3), forming two separate modules (Fig. 3b). Superposed by the MRG domains, the conformation of Cph1_PHD is almost identical to that of Cph2_PHD1, indicating that they likely play similar roles in the SIN3S complex (Fig. 3e). The Cph2_PHD1 and Cph1_PHD are conserved with the Rco1_PHD1, the PF1_PHD1, and other PHDs from BHC80, AIRE, and TRIM24 (Supplementary Fig. S11c), which enables their binding with the N-terminus of unmodified histone H3 of nucleosome^45^. The highly conserved residues Asp118 of Cph1 and Asp264 of Cph2 may recognize and bind to the residue Lys4 of the H3 histone (Supplementary Fig. S11d). Therefore, we christen these two modules “Histone Binding module 1/2” (HB1 or HB2), respectively. Besides, the N-terminus of Eaf3 contains a chromodomain (CHD) involved in the binding of methylated-H3K36^46–48^. Both CHDs of Eaf3 and Eaf3’ have no EM density captured in the current nucleosome-free state, likely due to their flexibility. Taken together, we consider that the SIN3S complex offers two sets of PHD-CHD to recognize the N-terminal tail of H3 histone with methylated Lys36.

Intriguingly, taking MRG domains as reference, the Cph2_PHD2 couldn’t be superposed to the BR domain of Cph1 (Fig. 3e). The Cph2_PHD2, located at the center of the structure of SIN3S, contributes to the stabilization and integrity of the whole complex. Besides, the N-terminal loop (residues 394–403) of the Cph2_PHD2 occupies the binding site of histone peptides in other PHDs (Supplementary Fig. S11d). It is consistent with the previous data that the human homolog PF1_PHD2 lacks histone H3 binding activity^25^. The BR domain, revealing a basic surface, is positioned at one corner of the SIN3S (Fig. 3b), likely to interact with the DNA or the acidic patch of the nucleosome.

### The interaction between Clr6 and the HID of Pst1/2/3

The histone deacetylase Clr6 belongs to the zinc-dependent class I HDAC family (Supplementary Fig. S12)^49^, which serves as the catalytic subunits of multiple transcriptional regulatory complexes^2,50^, such as SIN3, NuRD, CoREST, SMRT/NCoR and MiDAC. Compared with the available structural information of other class I HDAC complexes, our structures of SIN3L and SIN3S provide distinctive mechanisms for the HID of Pst1/2/3 to recruit the Clr6.

**Fig. 4:**
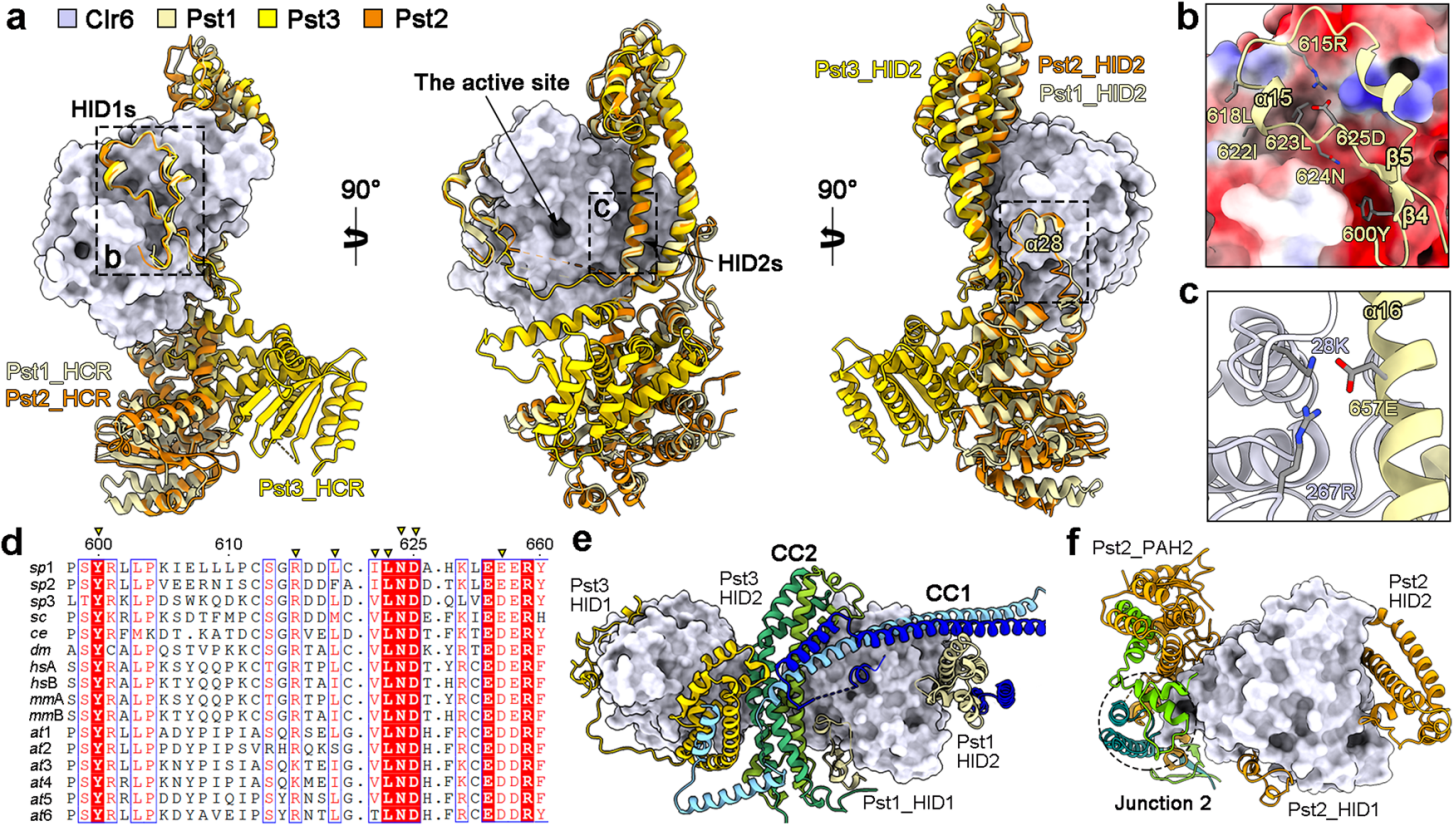
Stabilization of the Clr6 in the SIN3L or SIN3S complex. **a** The interactions between Clr6 and the HID of Pst1/2/3. The HID1 and HID2 interact with the Clr6 conservatively. The Clr6 is shown in surface, while Pst1, Pst2 and Pst3 are shown in cartoon. **b**, **c** Close-up views of the interface between Clr6 and HID1 (**b**) or HID2 (**c**). **d** The sequence alignment of Sin3 homologs from different species. *sp, Schizosaccharomyces pombe; sc, Saccharomyces cerevisiae; ce, Caenorhabditis elegans; dm, Drosophila melanogaster; hs, Homo sapiens; mm, Mus musculus; at, Arabidopsis thaliana*. **e**, **f** Clr6 is stabilized by the scaffold of the SIN3L (**e**) or the SIN3S (**f**) complex.

In the structures of SIN3L and SIN3S, the three molecules of Clr6 are almost identical (Supplementary Fig. S13a). By comparison among the Pst1/2/3 Lobes, two regions of HID (named HID1 and HID2) adopt highly conserved ways to interact with Clr6 (Fig. 4a–d). Taking Pst1–Clr6 as an example, the interactions involving the HID1 region include: the residue Tyr755 in the β4 inserts into the hydrophilic pocket of Clr6; the α15 (residues Leu618, Ile622, and Leu623) is embedded in the hydrophobic pocket of Clr6; and the residues Arg615, Asp625 of Pst1 and Lys141 of Clr6 form a hydrogen-bond network (Fig. 4a, b). In the HID2 region, the negatively charged residue Glu657 in the N-terminus of α16 interacts with the conserved positively charged pocket of Clr6 (Fig. 4a, c; Supplementary Fig. S13b). Notably, this pocket in the structures of other class I HDAC complexes (including HDAC1–MTA1^51^, HDAC1–MIDEAS^52^, and HDAC3–SMRT^53^ subcomplexes) is occupied by the inositol phosphates, which act as an ‘intermolecular glue’ that cements the HDAC and the SANT/DAD domain together (Supplementary Fig. S13b).

### Stabilization of Clr6 in the SIN3L or the SIN3S complex

The accommodation of Clr6 in the SIN3L and the SIN3S complexes is assisted not only by the HID1 and HID2 regions, but also by other structural features. The HCR_α22 of Pst1 or Pst2 directly interacts with Clr6, but this helix in Pst3 is shortened to a loop (Fig. 4a).

Beyond that, the scaffolds of these two complexes are also involved in the stabilization of the histone deacetylases. In SIN3L, the CC1 of the scaffold binds to the surface of Clr6 (Fig. 1c, right panel, Fig. 4e), and the CC2 directly connects Clr6 and Clr6’ to form an asymmetric dimer. This structural feature indicates that the stabilization of Clr6 and Clr6’ may influence each other in the SIN3L complex. While in SIN3S, Junction 2 of Cph1 and Cph2 serves as the third anchor (the other two refer to the HID1 and HID2 of Pst2) to stabilize the Clr6 (Fig. 4f).

### Structure comparison of the Pst1/3 Lobes in SIN3L and the Pst2 Lobe in SIN3S

**Fig. 5:**
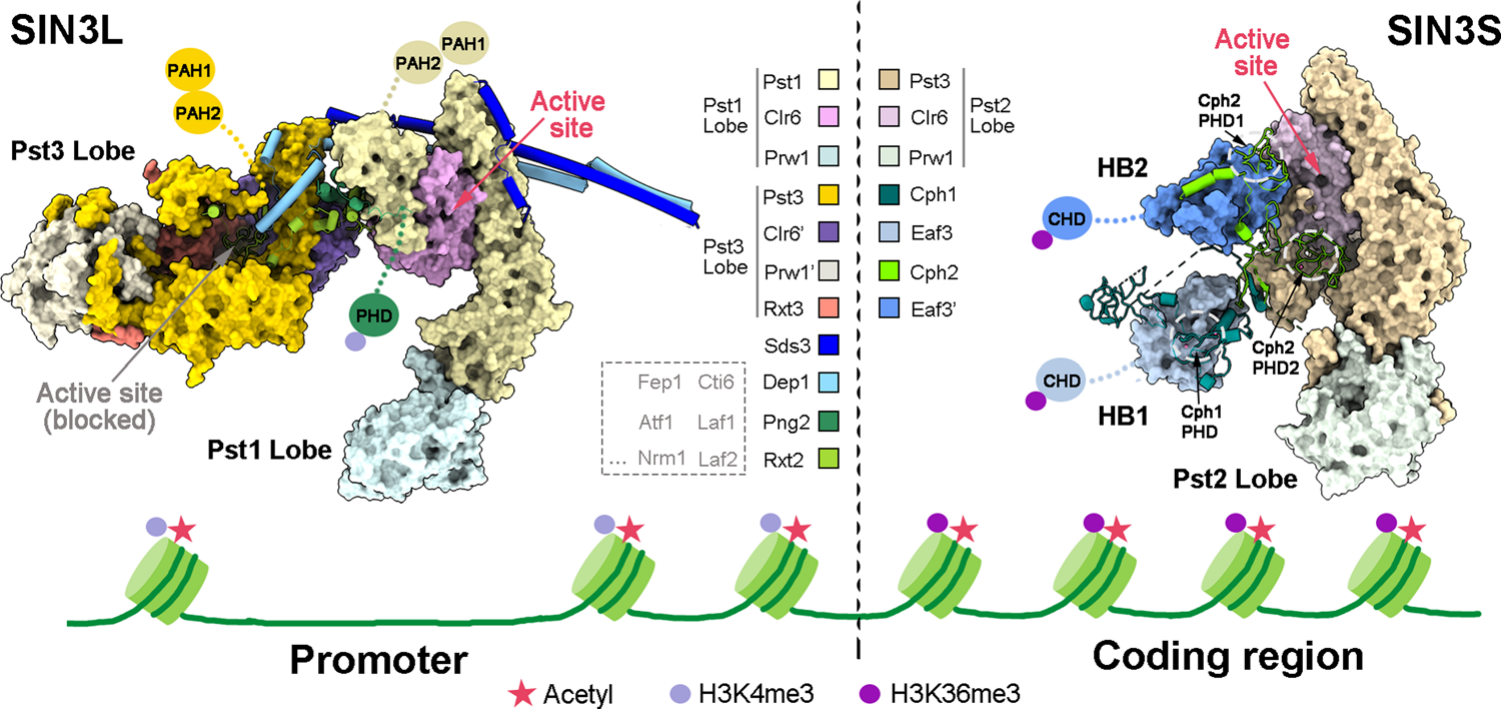
Two distinct assembly modes facilitate SIN3L and SIN3S to target different chromatin regions. The model shows that SIN3L and SIN3S adopt two distinct assembly modes to target different chromatin regions. The SIN3L complex has two lobes (Pst1/3 Lobe) bridged by two coiled-coil domains from Sds3/Dep1 and Rxt2/Png2, while the SIN3S complex has only one lobe (Pst2 Lobe) and two HBs (HB1 and HB2). The Pst1 Lobe in SIN3L and the Pst2 Lobe in SIN3S have similar conformations with their deacetylase active sites exposed to the space; the Pst3 Lobe in SIN3L is in a compact state with its active center buried inside and blocked. The SIN3L complex provides several domains for chromatin recognition, such as the PAH1/2 of Pst1/3 (interacting with transcription factors), the PHD of Png2 (may recognize the H3K4me3 histone marker), the PHD of Cti6 and the SWIRM domain of Laf1/2. Different combinations of these domains may be used to recognize specific promotors. The HB1 and HB2 of the SIN3S complex may offer two binding sites for the adjacent nucleosomes with H3K36me3 markers.

Sin3 protein is present as various isoforms in different species^54^. Unlike the budding yeast *S. cerevisiae* that has one isoform of Sin3 and mammals that have two (SIN3A and SIN3B), the fission yeast *S. pombe* has three (Pst1, Pst2, and Pst3). Our work first proved that these three isoforms performed non-redundant functions. They respectively form three distinct lobes (the Pst1/3 Lobes in the SIN3L complex, and the Pst2 Lobe in the SIN3S complex) (Fig. 5). Structure comparison among the three lobes reveals some similarities and remarkable differences. In each lobe, the Sin3 isoform (Pst1, Pst2, or Pst3) wraps up Clr6 through its HID and binds to the Prw1 through its HCR. The local interaction patterns between each HID and Clr6 are similar in the SIN3L/S complex, but different from other class I HDAC complexes (Fig. 4a; Supplementary Fig. S13b), providing potential targets for specific HDAC inhibitors design. Prw1 is the homolog of the *S. cerevisiae* Ume1 and the human RBBP4/7, containing an N-terminal NEE box and a C-terminal WD40 domain (Supplementary Fig. S14a). Both the NEE box and the WD40 repeat interact with the C-terminus of the Pst1/2/3 (Supplementary Fig. S14b), which is consistent with the previous genetic data^55^. In addition, the human RBBP7 was reported to interact with the unfolded helix-1 of histone H4^56^. However, superposing the structure of RBBP7 bound to histone H4 to the structure of the SIN3L or SIN3S complex, the helix-1 of histone H4 is clashed with one helix of the Pst1/2/3 protein (Supplementary Fig. S14c). Similarly, in the NuRD complex, this binding site of RBBP4 is occupied by one helix of MTA1^57,58^ (Supplementary Fig. S14d). It indicates that this interaction pattern is improper when RBBP4/7 or Prw1 serves as the component of the class I HDAC complex. At present, the function of Prw1 in the SIN3L/S complex remains elusive.

Furthermore, we realize that the Pst1/2/3 forms different conformations to create different “environments” for the active site. Notably, the overall conformation of the Pst1/2 Lobes are similar and extended with their adjacent active sites highly solvent accessible (Fig. 4a), suggesting that the Pst1 Lobe in SIN3L and the Pst2_Lobe in SIN3S play roles in histone deacetylation. In particular, the HCR and the PAH3 of Pst3 go through varying degrees of rotation to stabilize the Rxt2_NTD, which blocks the active site of Clr6’ (Figs. 4a, 5), implying the distinct function of the Pst3 Lobe in SIN3L. It is the first time to find that the histone deacetylase can be regulated by partners inside a multi-subunit complex. Further investigations are needed to determine whether this blocked active site could be open under certain circumstances or whether it just serves as a structural scaffold.

## Discussion

In this work we determined the atomic structures of the SIN3L and SIN3S complexes from *S. pombe*, revealing two representative organization mechanisms for the SIN3/HDAC complexes. Combining the structural analysis and previous reports, we find that the distinct assembly modes of SIN3L and SIN3S facilitate the HDACs to target different chromatin regions (Fig. 5). It is generally accepted that the SIN3L complex is recruited to the promoter region by DNA-binding elements and is essential for viability^11^. It is consistent with our mass spectrometry analysis that the endogenously purified SIN3L sample from the Pst3-Flag strain contains a set of DNA-binding proteins, including the iron-sensing transcriptional repressor Fep1, the start control protein Cdc10, the cell division cycle-related protein Res1/2, the pre-mRNA-processing factor Prp39, and other transcription factors (including Atf1, Nrm1, P14E8.02, etc.), which are absent in the SIN3S sample from the Pst2-Flag strain (Supplementary Tables S2, S3). In the SIN3L complex, the PAH1/2 domains of Pst1/3 are thought to interact with the transcription factors^1,59^. In addition, the SIN3L complex also provides other domains for chromatin recognition, such as the PHD of Png2 that may act as an epigenetic reader of the H3K4me3 histone (Supplementary Fig. S11b, c)^38,60^, the PHD of Cti6 and the SWIRM domain of Laf1/2. However, the EM density maps for these domains and the DNA-binding elements could not be clearly identified in SIN3L, suggesting that these domains or proteins are highly mobile. Considering the SIN3L complex as a general regulator of transcription^61^, different combinations of these chromatin-binding domains might take charge in recognizing the specific promotor. The flexibility between the chromatin-binding domain and the stable complex scaffold may provide the versatility of the SIN3L complex.

The yeast SIN3S complex is reported to recognize the H3K36me3 marker and function in the gene coding regions for suppression of antisense transcription and protection from genotoxic agents^10^. The PAH1/2 domains of the Pst2, different from that of the Pst1/3, are well identified to interact with Cph1 and Cph2, connecting the HB1 and HB2 to the Pst2 Lobe (Fig. 3a). In the structure of SIN3S, we find that the HB1 and HB2 adopt different orientations. The Cph2_PHD1 in HB2 is adjacent to the active site; while the distance between the Cph1_PHD in HB1 and the active site is as much as 80 Å in length (Fig. 5; Supplementary Fig. S11b). It was realized that the *S. cerevisiae* SIN3S (also called Rpd3S) complex preferentially bound di-nucleosomes^18^. Therefore, we suppose that the HB1 and HB2 of the SIN3S complex may offer two binding sites for the adjacent nucleosomes. It needs further studies to confirm.

Furthermore, two deacetylases were previously suggested to be needed for the class I HDAC complexes (including SIN3, NuRD, SMART, CoREST, MiDAC, etc.)^62^, and two HDAC molecules were found to be symmetric in the structures of the human NuRD subcomplex^51^ and the MiDAC subcomplex^51,52^. We show in this study that the SIN3L complex contains two asymmetric deacetylase active sites with one open and the other blocked, and the SIN3S complex only has one. These findings shed new light on the diverse and complex molecular mechanisms for the class I HDAC complexes.

In addition, based on the sequence conservation analysis (Supplementary Figs. S9, S12, and S14a), we mapped a subset of invariant residues from the human cancer mutation database onto our SIN3L/SIN3S models^63^. The majority of the mutations probably compromise the folding of the structure, and many other mutations are located at the HDAC active site, the hydrophobic pocket of the PAH domains, the Sin3–HDAC interface, and the Sin3–Prw1 interface (Supplementary Fig. S15). In summary, the advent of structures of the SIN3L and the SIN3S from *S. pombe* offers a framework for studying and understanding the mechanisms and functions of the SIN3/HDAC complexes.

## Materials and methods

### The *S. pombe* strain

The *S. pombe* strain used to purify the SIN3L or SIN3S complex carries a 3× Flag-tag (DYKDHDGDYKDHDIDYKDDDDK) at the C-terminus of the Sin3 homolog Pst3 or Pst2. The DNA sequences for the 3× Flag-tag and a HphMX6 marker were amplified by PCR from the plasmid pF6Aa-C3Flag-HphMX6. The PCR product was transformed into a wild-type *S. pombe* strain (kindly provided by Dr. Rui Bai) by the lithium acetate method^66^. Transformants were selected on hygromycin containing 2× YES solid medium. Correct integration of the 3× Flag-tag was further confirmed by PCR and western blotting.

### Purification of the SIN3L and SIN3S complexes

The Pst3-tagged or Pst2-tagged culture was grown on 1× YES medium for 8–10 h at 30 °C to an OD_600_ of ~6. The collected cell pellets were washed with buffer containing 1 mM phenylmethylsulfonyl fluoride (PMSF), and resuspended in lysis buffer containing 50 mM HEPES (pH 7.4), 350 mM NaCl, 15% glycerol (v/v) and protease inhibitors (1 mM PMSF, 2.6 μg/mL aprotinin, 1.4 μg/mL pepstatin, and 5 μg/mL leupeptin). The cell suspension was dropped into liquid nitrogen to form yeast beads and pulverized to powder in a Retsch ZM200 nitrogen mill. The frozen yeast powder was thawed at room temperature and resuspended in the same lysis buffer. The cell lysate was first centrifuged at 12,000 rpm for 30 min and the supernatant was centrifuged again at 12,000 rpm for another 1 h. The resulting supernatant was loaded into the ANTI-FLAG M2 resin (Sigma) and eluted using eluate buffer containing 0.5 mg/mL FLAG peptide (DYKDDDDK), 20 mM HEPES (pH 7.4), 150 mM NaCl, 5% glycerol (v/v). The eluate was concentrated and then applied for glycerol density gradient centrifugation. The glycerol gradient was prepared with light-buffer containing 10% glycerol (v/v), 20 mM HEPES (pH 7.4), 50 mM NaCl, 2 mM DTT, and heavy-buffer containing 30% glycerol (v/v), 20 mM HEPES (pH 7.4), 50 mM NaCl, 2 mM DTT. After centrifugation at 30,000 rpm for 20 h at 4 °C using a Beckman SW32Ti rotor, the peak fractions containing SIN3L or SIN3S complex were verified by SDS-PAGE (Supplementary Fig. S1a, b), and further cross-linked by 1 mM BS3 on ice for 2 h and quenched by 50 mM Tris-HCl (pH 7.4), followed by dialysis for 12 h against the buffer containing 20 mM HEPES (pH 7.4), 50 mM NaCl, and 2 mM DTT.

### Mass spectrometry analysis

The SDS-PAGE was used to separate the purified SIN3L or SIN3S complex and stained it with Coomassie Blue G-250. The gel bands of interest were cut into pieces. Samples were digested by trypsin with prior reduction and alkylation in 50 mM ammonium bicarbonate at 37 °C overnight. The digested products were extracted twice with 1% formic acid in 50% acetonitrile aqueous solution and dried to reduce volume by SpeedVac.

For LC-MS/MS analysis, the peptides were separated by a 65 min gradient elution at a flow rate 0.300 µL/min with the Thermo EASY-nLC1200 integrated nano-HPLC system which is directly interfaced with the Thermo Q Exactive HF-X mass spectrometer. The analytical column was a home-made fused silica capillary column (75 µm ID, 150 mm length; Upchurch, Oak Harbor, WA) packed with C-18 resin (300 A, 3 µm, Varian, Lexington, MA). Mobile phase A consisted of 0.1% formic acid, and mobile phase B consisted of 100% acetonitrile and 0.1% formic acid. The mass spectrometer was operated in the data-dependent acquisition mode using the Xcalibur 4.1 software and there is a single full-scan mass spectrum in the Orbitrap (300–1800 m/z, 60,000 resolution) followed by 20 data-dependent MS/MS scans at 30% normalized collision energy. Each mass spectrum was analyzed using the Thermo Xcalibur Qual Browser and Proteome Discovery for database searching.

### Sample preparation and EM data collection

Uranyl acetate (2% w/v) was used for negative staining. Briefly, 4-μL aliquots of the cross-linked sample at the concentration of 0.02 mg/mL were applied to the glow-discharged copper grid supported by a thin layer of carbon film (Zhongjingkeyi Technology Co., Ltd) for 1 min. The negatively stained samples were imaged on a Thermo Fisher Talos L120C TEM microscope operating at 120 kV. The glow-discharged copper Lacey carbon grids (TED PELLA) were used for cryo-EM specimen preparation. Cryo-EM grids were prepared using Vitrobot Mark IV (FEI Company) operating at 8 °C and 100% humidity. After waiting 1 min, 4-μL sample at a 0.1 mg/mL concentration was blotted and rapidly plunged into liquid ethane cooled by liquid nitrogen.

Cryo-EM specimens were imaged on a 300-kV Titan Krios electron microscope (Thermo Fisher Scientific) with a normal magnification of ×81,000. Movies were collected by a Gatan K3 direct electron detector equipped with a GIF Quantum energy filter (slit width 20 eV) at the super-resolution mode. The micrographs were automatically recorded using EPU (Thermo Fisher Scientific) with a defocus range from −1.8 μm to −2.3 μm. Each stack of 32 frames was exposed for 2.56 s with total dose of ~50 e^−^/Å^2^, and aligned and summed using MotionCor2 with a binning factor of 2, resulting in a pixel size of 1.087 Å^67^. Dose weighting was performed concurrently. The defocus value for each image was determined by Gctf^68^.

### Cryo-EM data processing

The simplified cryo-image data processing procedures for the SIN3L or SIN3S complex can be found in Supplementary Fig. S2. For the SIN3L complex, all steps were mainly carried out using RELION 3.0^69^ except that is specially mentioned. In total, ~2.1 millions particles were generated from 7294 micrographs using Gautomatch (developed by Kai Zhang, https://www.mrc-lmb.cam.ac.uk/kzhang/Gautomatch/). The generation of initial 3D volume of the SIN3L complex was from preliminary data analysis using cryoSPARC v3^70^. Single-reference and multi-reference guided 3D-classifications were applied to the total particles, and about 562 thousand good partilces were selected. For further processing, local masks were applied to different parts of the initial reconstruction. Finally, about 263 and 389 thousands select partilces yielded the reconstructions at average resolutions of 4.0 Å and 3.2 Å after refinement for the Prw1 and main regions, respectively (Supplementary Fig. S2a).

For the SIN3S complex, all the precessing were performed in cryoSPARC v3^70^. Similarly, about 1.4 million particles were auto-picked from 3523 micrographs. Two rounds of 2D classificaion gave rise to a data set containing about 860 thousand good particles. After initial reference generation and further hetero-refinement, a final reconstruction at 2.9 Å was obtained from about 777 thousand particles using NU-refinement (Supplementary Fig. S2b).

Reported resolutions mentioned above were calculated on the basis of the Fourier shell correlation (FSC) 0.143 criterion^71^ (Supplementary Fig. S3a). Prior to visualization, all EM maps were postprocessed and sharpened by applying a negative B-factor for SIN3L and SIN3S in RELION^69^ and cryoSPARC^70^, respectively. Local resolution variations were estimated for SIN3L and SIN3S using ResMap^72^ and cryoSPARC^70^, respectively (Supplementary Fig. S3b, c and Table S1). The angle distributions of the particles used in the final reconstructions are reasonable (Supplementary Fig. S3d, e).

### Model building and refinement

The atomic models of the *S. pombe* SIN3L and SIN3S complexes were built de novo based on our high-resolution EM maps using COOT^73^ (Supplementary Table S1). We first placed the poly-Ala sequences into the EM maps and successfully assigned the different components under the guidance of the individual predicted structure from the AlphaFold database^74^. Then the individual reliable domains of the predicted structures of each component were fitted into the EM maps using Chimera^75^, and manually adjusted in COOT. The linker sequences were built de novo based on the clear features of bulk residues.

The final atomic coordinates of the *S. pombe* SIN3L and SIN3S complexes were respectively refined according to the 3.2-Å and 2.9-Å EM maps using PHENIX in real space^76^ and secondary structure restraints that were generated meanwhile. Overfitting of the model was monitored by refining the model in one of the two independent maps from the gold-standard refinement approach, and testing the refined model against the other^77^ (Supplementary Fig. S3f, g). The structures of the SIN3L and SIN3S complexes were validated through examination of the Molprobity scores and statistics of the Ramachandran plots (Supplementary Table S1). Molprobity scores were calculated as described^78^.

## Supplementary information

Supplementary materials

Supplementary Table S2

Supplementary Table S3

## Data Availability

The atomic coordinates for the *S. pombe* SIN3L and SIN3S complexes have been deposited in the Protein Data Bank with the accession code 8I03 and 8I02, and the EM maps have been deposited in EMDB with the accession code EMD-35093, EMD-35094, and EMD-35095 for SIN3L, and EMD-35092 for SIN3S.
