# Supplementary material for: Two assembly modes for SIN3 histone deacetylase complexes
Source: Cell Discov. 2023 Apr 19;9:42. doi: 10.1038/s41421-023-00539-x (PMC10115800; doi:10.1038/s41421-023-00539-x)
Supplement: Supplementary file 1 — Supplementary materials [file 41421_2023_539_MOESM1_ESM.pdf]

Supplementary Information for

Two assembly modes for SIN3 histone deacetylase complexes

Authors:

Chengcheng Wang[1,2,3,*,†], Zhouyan Guo[1,2,3,*], Chen Chu[1,2,3,*], Yichen Lu[1,2,3], Xiaofeng Zhang[1,2,3], and Xiechao Zhan[1,2,3,†].

Affiliations:

[1]Westlake Laboratory of Life Sciences and Biomedicine, 18 Shilongshan Road, Hangzhou 310024, Zhejiang Province, China

[2]Key Laboratory of Structural Biology of Zhejiang Province, School of Life Sciences, Westlake University; [3]Institute of Biology, Westlake Institute for Advanced Study; 18 Shilongshan Road, Hangzhou 310024, Zhejiang Province, China

[*]These authors contributed equally to this work.

†Corresponding authors. E-mail: wangchengcheng@westlake.edu.cn (C. Wang) & zhanxiechao@westlake.edu.cn (X. Zhan)

This file includes:

Supplementary Figures S1 to S15

Supplementary Table S1

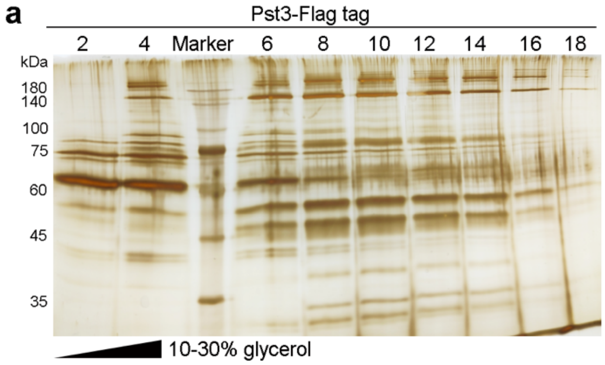

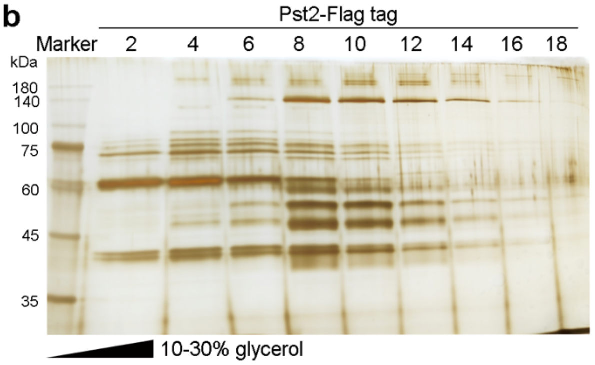

c

Accession	Subunit	Sum PEP Score	Coverage [%]	# PSMs	# Unique Peptides	MW [kDa]
Q09750	Pst1	1056.1	70	570	117	171.4
O74755	Pst3	988.0	79	695	91	132.8
O14021	Prw1	386.9	84	217	29	48.4
Q9P7M1	Dep1	299.9	70	126	35	55.6
O74736	Png2	283.2	86	80	28	34.9
O59702	Clr6	271.7	63	175	26	46.1
Q1MTR4	Cti6	246.0	58	62	23	46.9
O94707	Rxt3	221.4	74	73	22	39.4
Q9UTB6	Sds3	196.9	67	55	21	30.7
O13719	Laf1	130.6	68	51	21	33.9
O74443	Laf2	74.0	47	36	14	31.1
O94355	Rxt2	58.5	34	48	8	27.1

d

Accession	Subunit	Sum PEP Score	Coverage [%]	# PSMs	# Unique Peptides	MW [kDa]
O13919	Pst2	714.8	70	631	106	124.8
Q09698	Cph2	321.4	64	148	35	68.7
O14021	Prw1	262.7	75	150	28	48.4
O13953	Eaf3	258.2	69	201	27	39.1
O59702	Clr6	221.4	49	104	20	46.1
Q09819	Cph1	198.3	53	108	22	45.0

Supplementary Fig. S1 Purification and characterization of the SIN3L and SIN3S complexes from *S. pombe*.

a, The peak fractions of the SIN3L complex from glycerol density gradient centrifugation were visualized on SDS-PAGE by silver staining. Fractions 8-14 were collected for cryo-EM sample preparation. **b**, The peak fractions of the SIN3S complex from glycerol density gradient centrifugation were visualized on SDS-PAGE by silver staining. Fractions 8-12 were collected for cryo-EM sample preparation. **c-d**, Protein components of the SIN3L complex (**c**) or the SIN3S complex (**d**) were confirmed by mass spectrometry analysis.

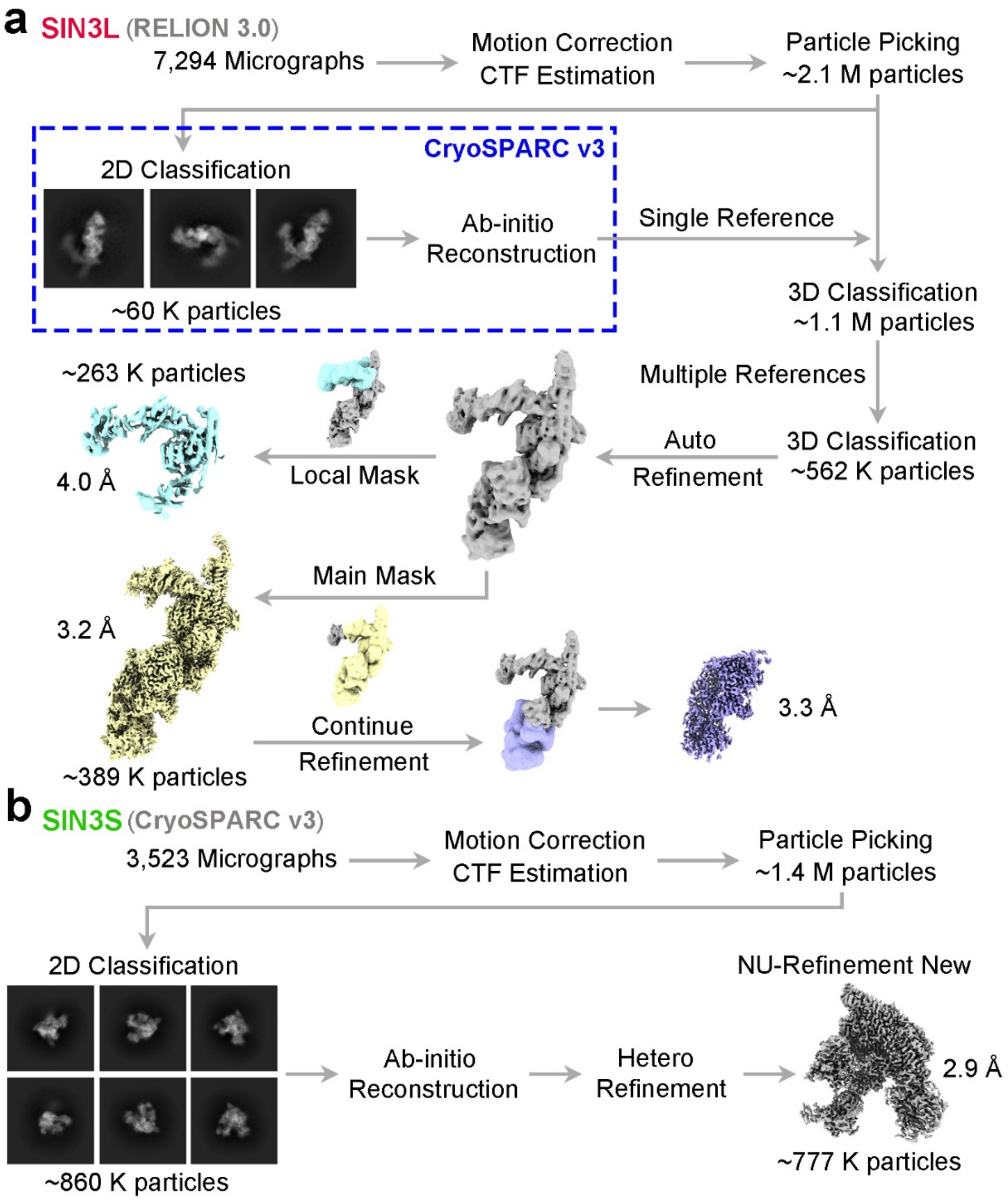

Supplementary Fig. S2 Flowcharts of the EM data processing for the SIN3L and SIN3S complexes.

a, A flowchart of the EM data processing for the SIN3L complex. All steps were carried out in RELION 3.0 and cryoSPARC v3. **b**, A flowchart of the EM data processing for the SIN3S complex. All steps were carried out in cryoSPARC v3. Please refer to Methods for details.

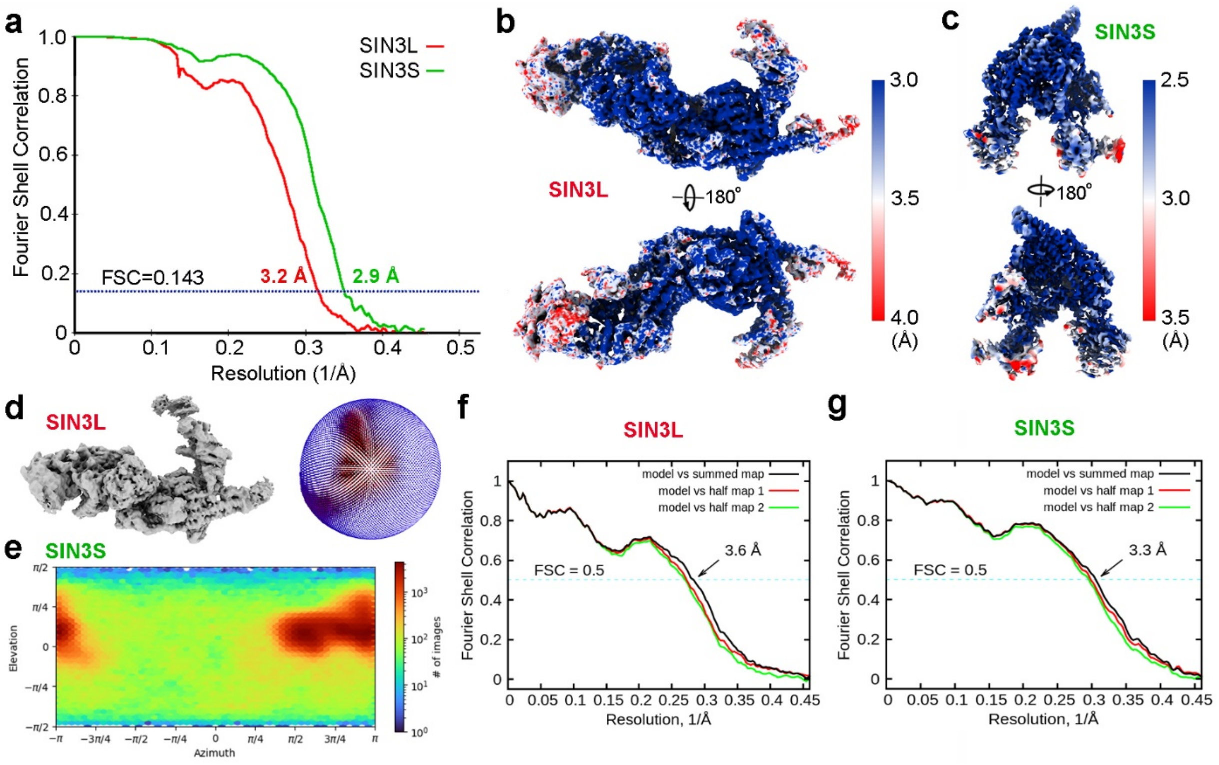

Supplementary Fig. S3 Cryo-EM analysis of the SIN3L and SIN3S complexes.

a, The final reconstructions for the SIN3L (red line) and SIN3S (green line) complexes display average resolutions of 3.2 and 2.9 Å on the basis of the Fourier-shell correlation (FSC) value of 0.143, respectively. **b-c**, Two related views of the EM density map for the SIN3L complex (**b**) and the SIN3S complex (**c**). The local resolutions of the EM density maps are color-coded. **d-e**, Angular distribution of the particles used in the final reconstructions for SIN3L in RELION 3.0 (**d**) and SIN3S in cryoSPARC v3 (**e**). **f-g**, The FSC curves for cross-validation between the models and the EM density maps for SIN3L (**f**) and SIN3S (**g**). Shown here are the FSC curves between the final refined atomic models and the reconstructions from all particles (black), between the respective model refined in the reconstruction from only half of the particles and the reconstruction from that same half (red), and between that same model and the reconstruction from the other half of the particles (green).

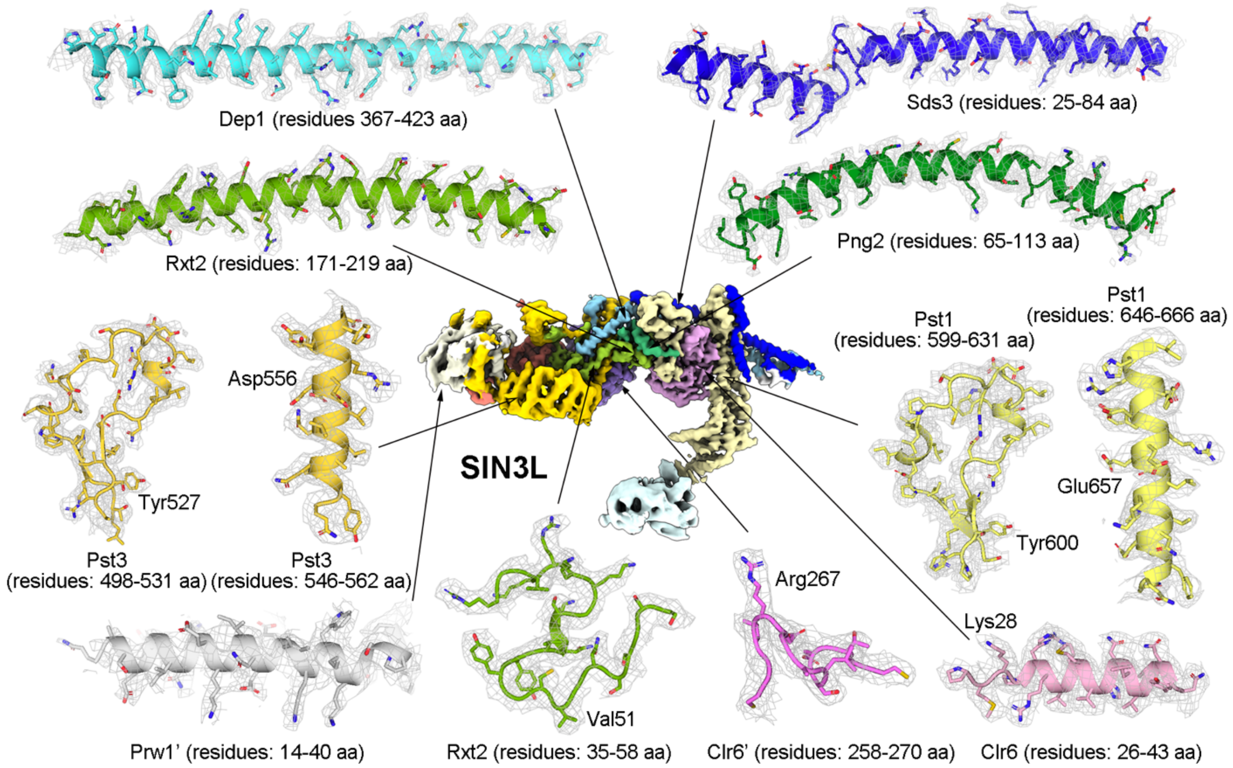

Supplementary Fig. S4 Representative EM density maps of the SIN3L complex.

Close-up views for representative fragments of the colored SIN3L subunits with EM densities shown as gray meshes. The side chains of representative bulk residues shown as sticks that are used to validate the sequence assignment are labeled. The EM maps were shown in PyMOL and contoured at 5 σ.

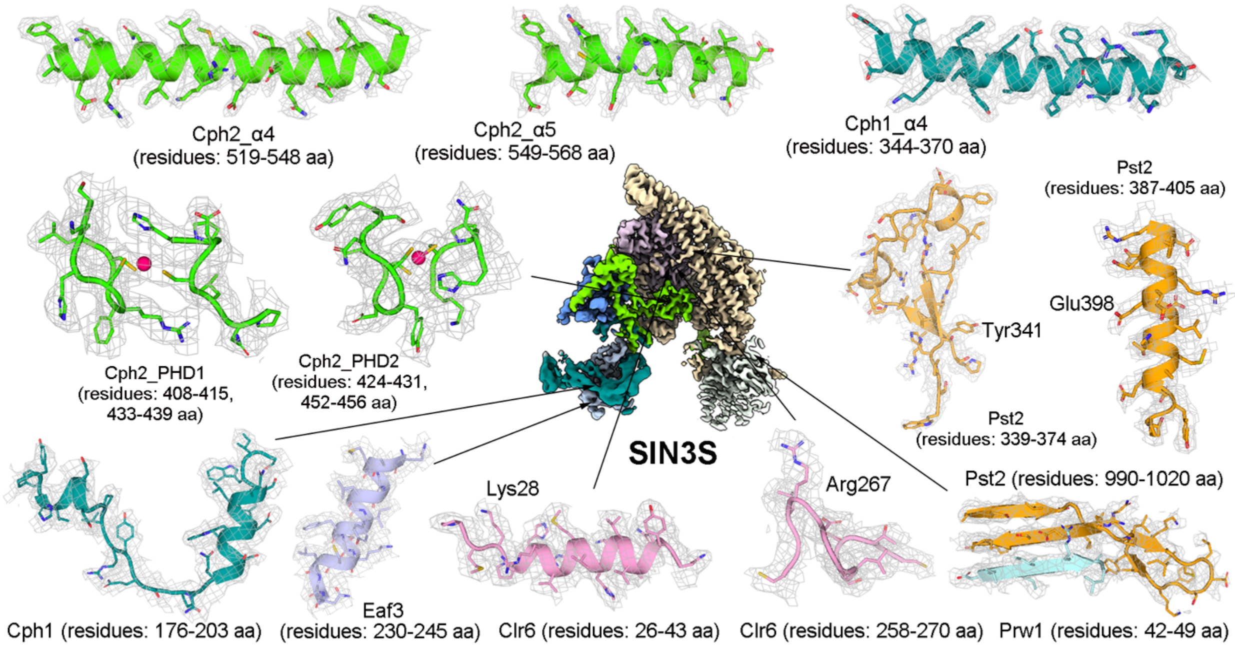

Supplementary Fig. S5 Representative EM density maps of the SIN3S complex.

Close-up views for representative fragments of the colored SIN3S subunits with EM densities shown as gray meshes. The side chains of representative bulk residues shown as sticks that are used to validate the sequence assignment are labeled. The EM maps were shown in PyMOL and contoured at 5 σ.

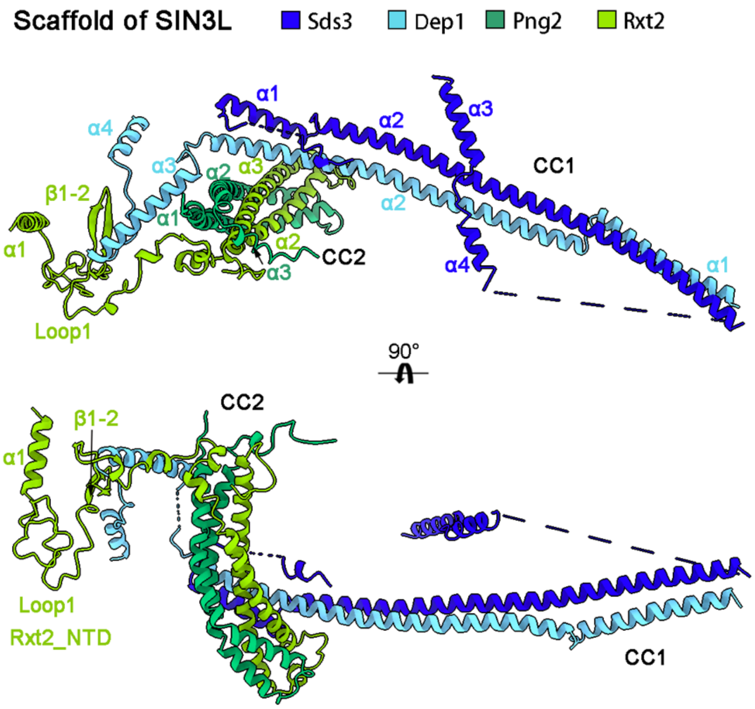

Supplementary Fig. S6 The scaffold of the SIN3L complex.

Two perpendicular views for the scaffold of the SIN3L complex. The Sds3/Dep1 and Rxt2/Png2 provide two coiled-coil domains (CC1 and CC2) to form the scaffold. The Sds3, Dep1, Png2 and Rxt2 are shown as cartoon and colored individually. The CC1 is made up of Sds3_α1-3 and Dep1_α1-3; the CC2 is formed by Rxt2_α1-3 and Png2_α1-3.

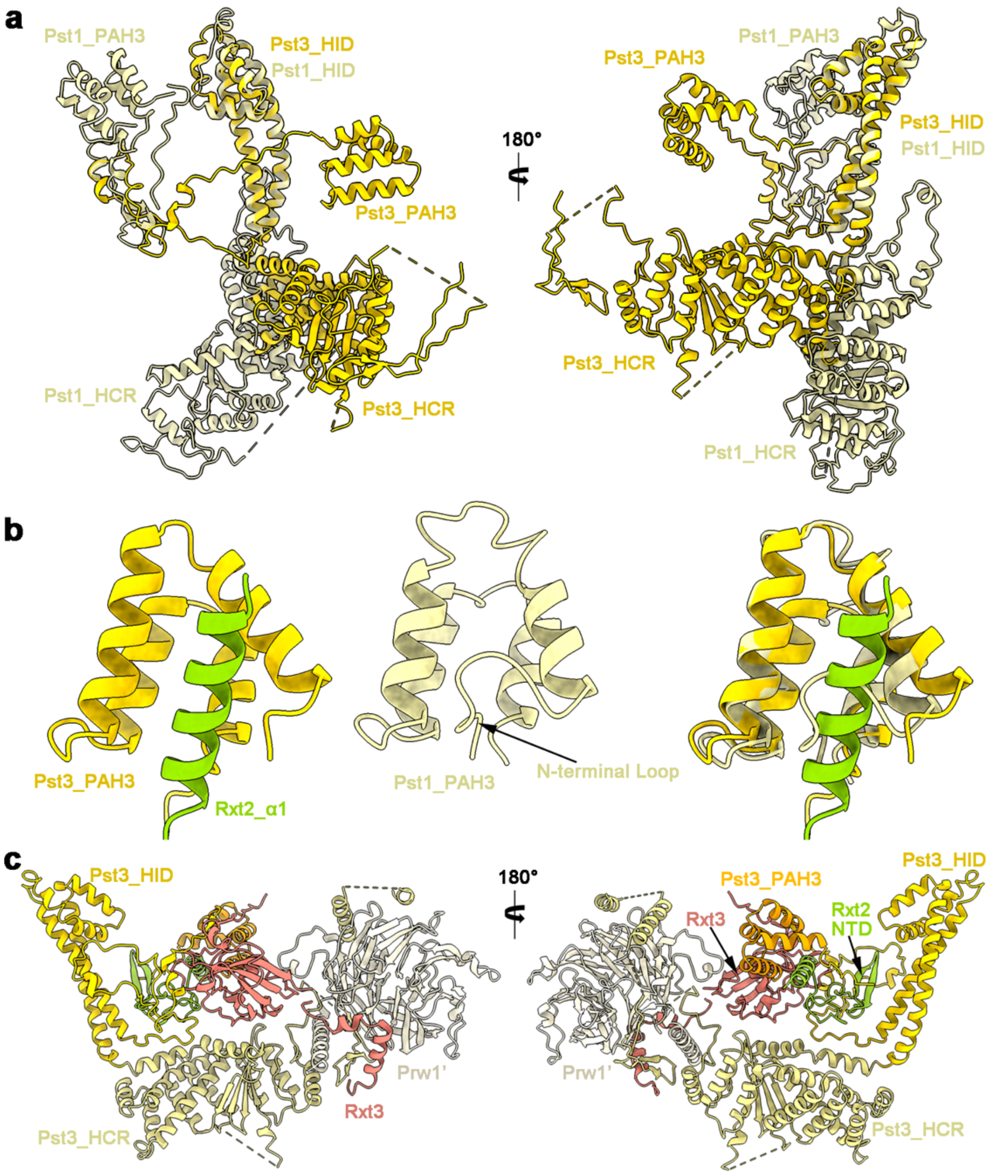

Supplementary Fig. S7 Structure comparison between the Pst1 and Pst3.

a, Two related views for the structures of the Pst1 and Pst3 superposed by the respective HID. **b**, The structures of the Pst1_PAH3 and the Pst3_PAH3. The Pst3_PAH3 associates with Rxt2_α1, while the Pst1_PAH3 interacts with its own N-terminal loop. **c**, Two related views for the interactions between Pst3_HCR, Prw1', and Rxt3.

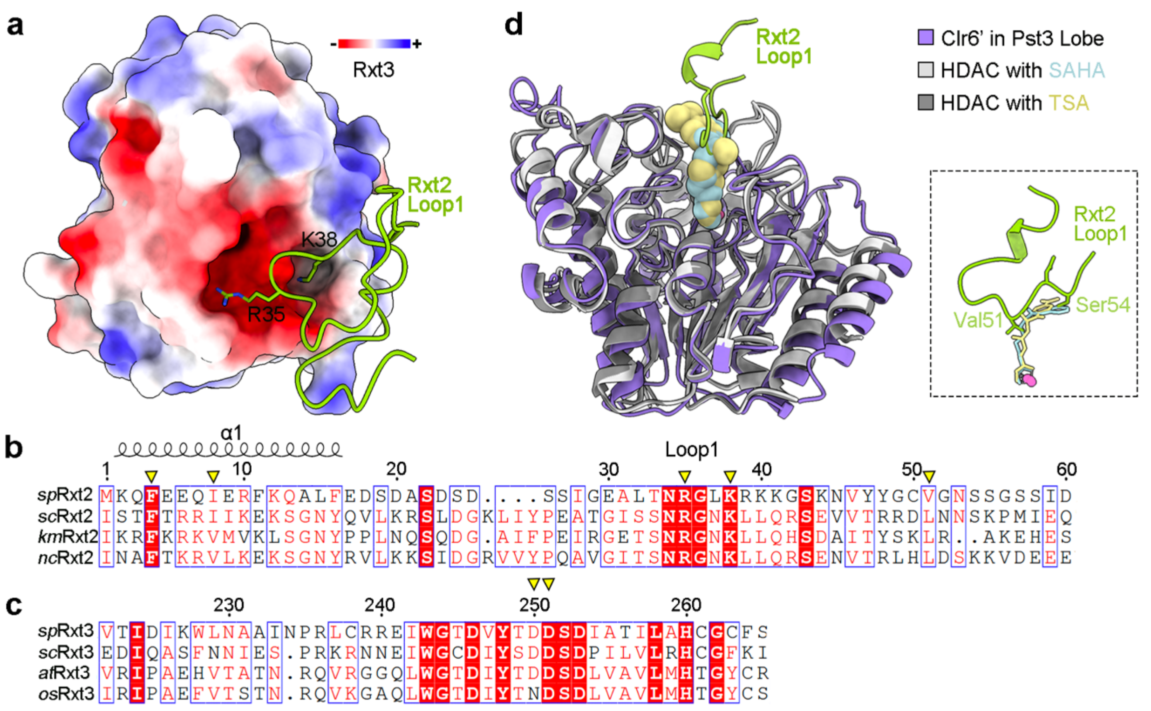

Supplementary Fig. S8 The Rxt2_loop1 is stabilized by Rxt3 and blocks the active center of Clr6'.

a, The interface between the Rxt2_loop1 and Rxt3. The Rxt3 is shown as electrostatic potential surface. **b**, The sequence alignment of the Rxt2_NTD from different species. The secondary structure elements modeled in the SIN3L complex are indicated above the sequences. *sp, Schizosaccharomyces pombe; sc*, *Saccharomyces cerevisiae*; *km*, *Kluyveromyces marxianus*; *nc*, *Naumovozyma castellii.* **c**, The sequence alignment of Rxt3 from different species. Rxt3-like proteins have been found in fungi and plants. *sp, Schizosaccharomyces pombe; sc, Saccharomyces cerevisiae; at, Arabidopsis thaliana; os, Oryza sativa.* **d**, The superposition of the structures of the Rxt2-Clr6 subcomplex and the bacterium HDAC bound to the inhibitors TSA (dark grey, PDB code: 1C3R) or SAHA (grey, PDB code: 1C3S). The residues 51-54 of the Rxt2 occupy the binding site of the TSA or SAHA molecule.

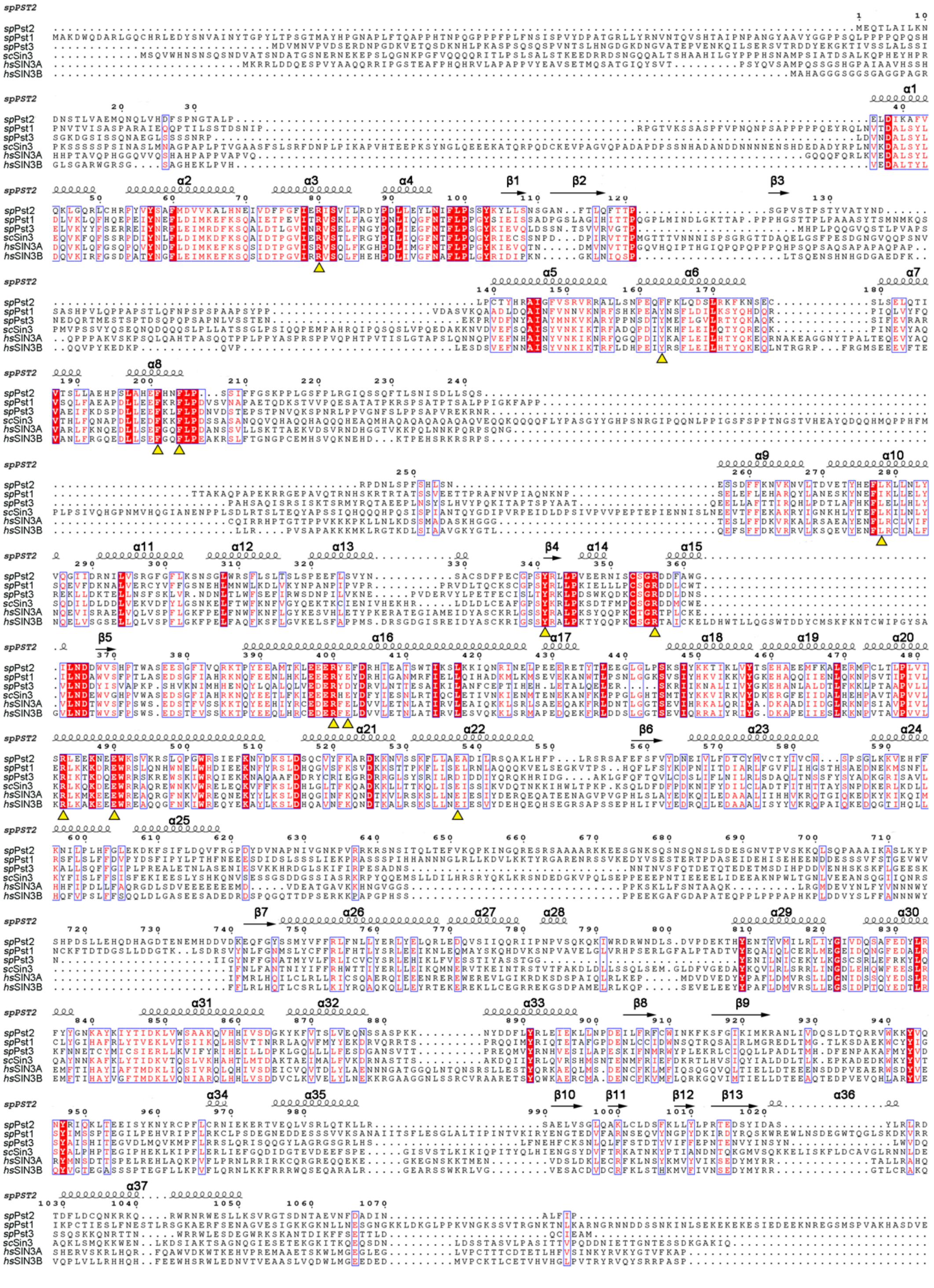

Supplementary Fig. S9 The sequence alignment of Sin3 homologs from different species.

The secondary structure elements of *sp*Pst2 modeled in the SIN3S complex are indicated above the sequences. *sp, Schizosaccharomyces pombe; sc*, *Saccharomyces cerevisiae*; *hs, Homo sapiens.* Yellow triangles depict the invariant residues harboring mutations from patients with cancer that occur at interfaces between conserved subunits.

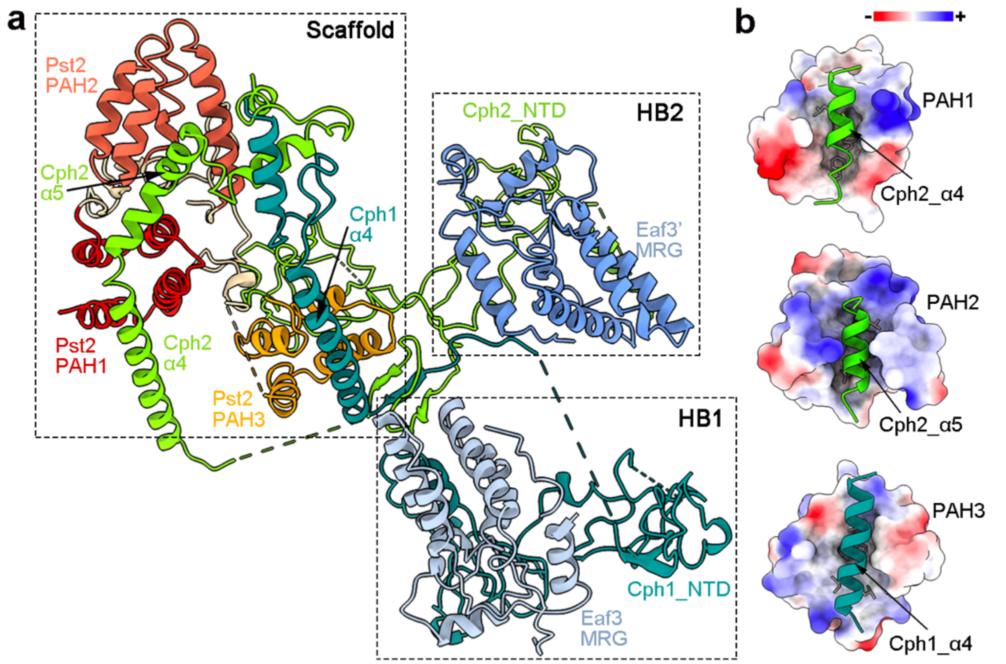

Supplementary Fig. S10 The Cph1 and Cph2 play essential roles in the stabilization of the SIN3S complex.

a, The CTDs of Cph1 and Cph2 form the scaffold with the PAH1/2/3 of Pst2. The NTDs of Cph1 and Cph2 recruit the MRG domains of Eaf3 and Eaf3' through their SID domains (α2-3), forming the HB1 and HB2 modules, respectively. **b**, The PAH1/2/3 of Pst2 adopt different conformations to stabilize the Cph2_ α4, Cph2_α5, or Cph1_α4 in the back side of the deacetylase activity center, respectively.

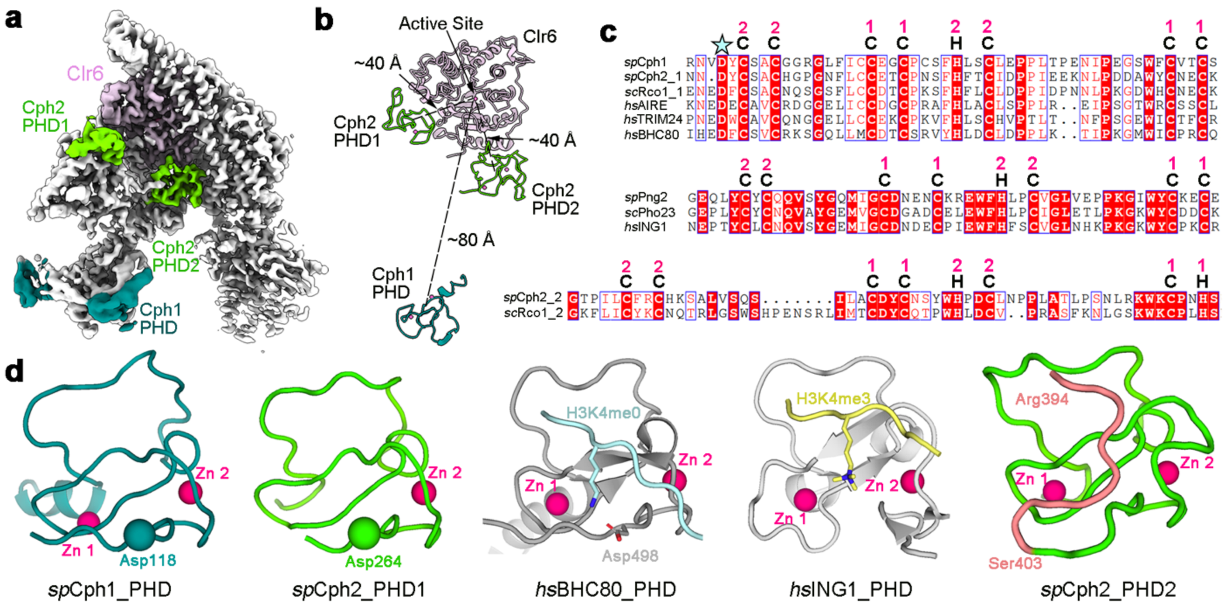

Supplementary Fig. S11 The three PHDs of the Cph1 and Cph2 in the SIN3S complex.

a, The localization of the three PHDs in the SIN3S complex. Shown here is the EM density map of SIN3S with three PHDs color-coded. **b**, Structure features of the three PHDs. **c**, The sequence alignment of the different types of the PHDs. The Cph2_PHD1 and Cph1_PHD are conserved with the Rco1_PHD1 and other PHDs from the BHC80, AIRE, and TRIM24, which allow their binding with the unmodified N-terminus of the H3. The Png2 belongs to the inhibitor of growth (ING) family and contains a C-terminal conserved PHD, which serves as an epigenetic reader of the H3K4me3 histone mark. The Cph2_PHD2 is conserved with the Rco1_PHD2, which cannot be categorized as the types which recognize H3K4me2/3 or H3K4me0. Zinc-coordinating residues are indicated as uppercase letters above the sequences. *sp, Schizosaccharomyces pombe; sc, Saccharomyces cerevisiae; hs, Homo sapiens*. **d**, The structures of different PHDs. Corresponding to the structure of human BHC80_PHD bound with the unmodified N-terminus of the H3, The residue (Glu118 in Cph1_PHD, and Glu264 in Cph2_PHD1) may associate with the H3K4me0. The N-terminal loop (residues 394-403) of the Cph2_PHD2 occupies the binding site of histone peptides in other PHDs.

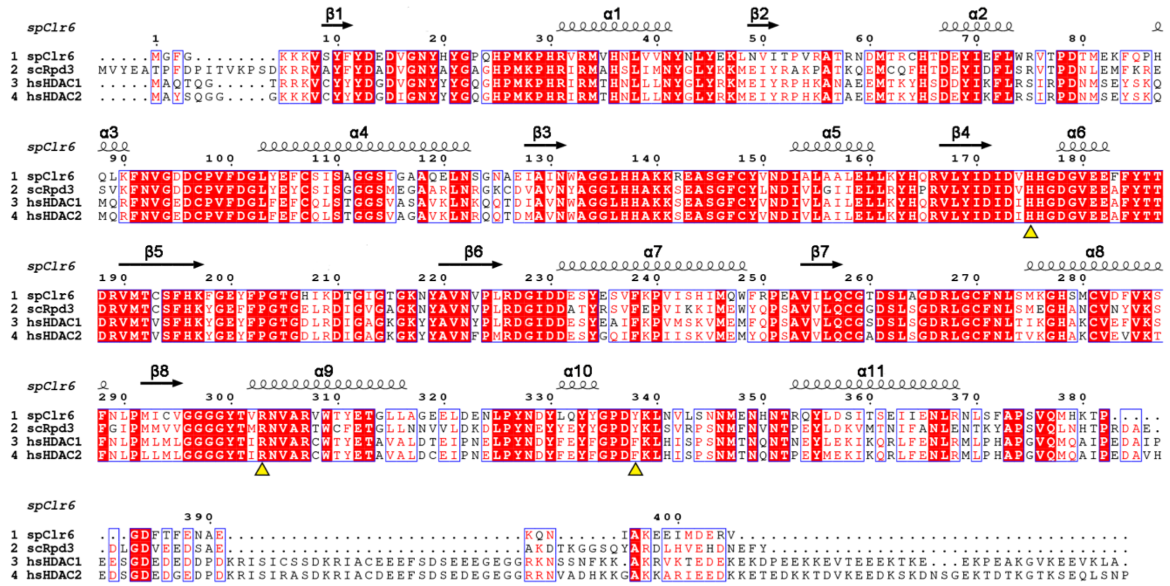

Supplementary Fig. S12 The sequence alignment of Clr6 homologs from different species.

The secondary structure elements of *sp*Clr6 modeled in the SIN3S complex are indicated above the sequences. *sp, Schizosaccharomyces pombe; sc*, *Saccharomyces cerevisiae*; *hs, Homo sapiens.* Yellow triangles depict the invariant residues harboring mutations from patients with cancer that occur at interfaces between conserved subunits.

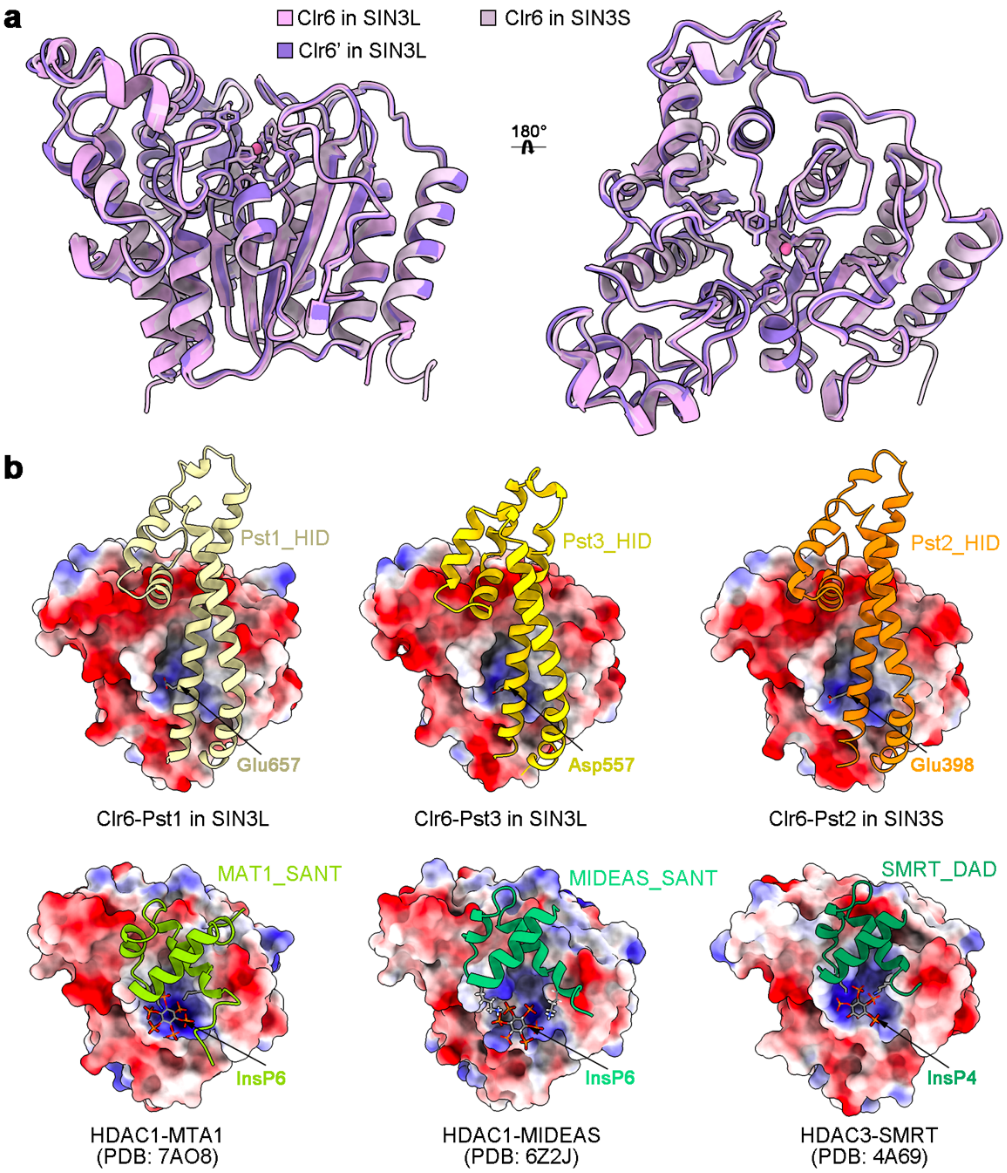

Supplementary Fig. S13 The structures of the class I HDACs.

a, The three molecules of Clr6 in the SIN3L and SIN3S complexes are almost identical. **b**, The negatively charged residue (Glu657 in Pst1, Asp557 in Pst3, and Glu398 in Pst2) interacts with the conserved positively charged pocket of the Clr6. Notably, this pocket in the structures of other class I HDAC complexes (including HDAC1-MTA1, HDAC1-MIDEAS, and HDAC3-SMRT subcomplexes) is occupied by the inositol phosphates, which acts as ‘intermolecular glue’ that cement the HDAC and the SANT/DAD domain together.

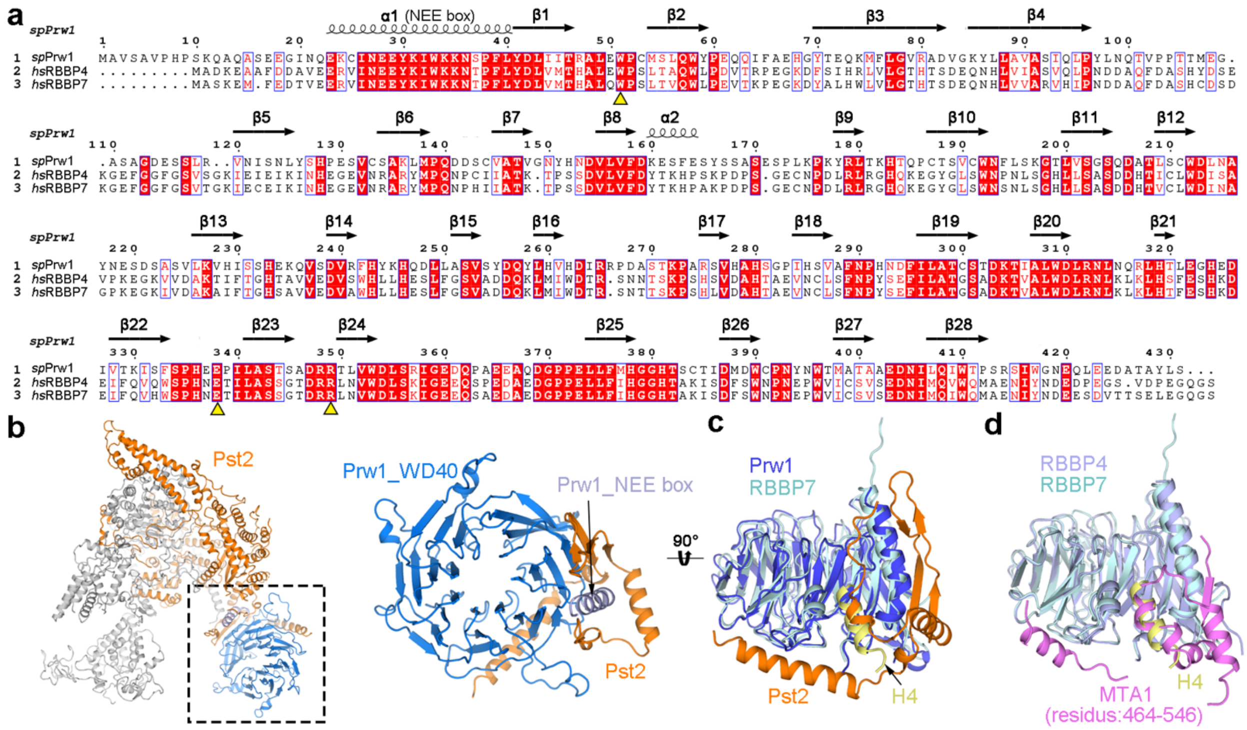

Supplementary Fig. S14 The interaction pattern for the WD40-containing proteins.

a, The sequence alignment of Prw1 homologs from different species. The secondary structure elements of *sp*Prw1 modeled in the SIN3S complex are indicated above the sequences. *sp, Schizosaccharomyces pombe; hs, Homo sapiens.* Yellow triangles depict the invariant residues harboring mutations from patients with cancer that occur at interfaces between conserved subunits. **b,** The interaction between the Prw1and Pst2 in the SIN3S complex. **c**, Structure superposition between the Prw1-Pst2 and the RBBP7-H4 (PDB code: 3CFV). **d**, Structure superposition between the RBBP4-MTA1 (PDB code: 5FXY) and the RBBP7-H4 complex.

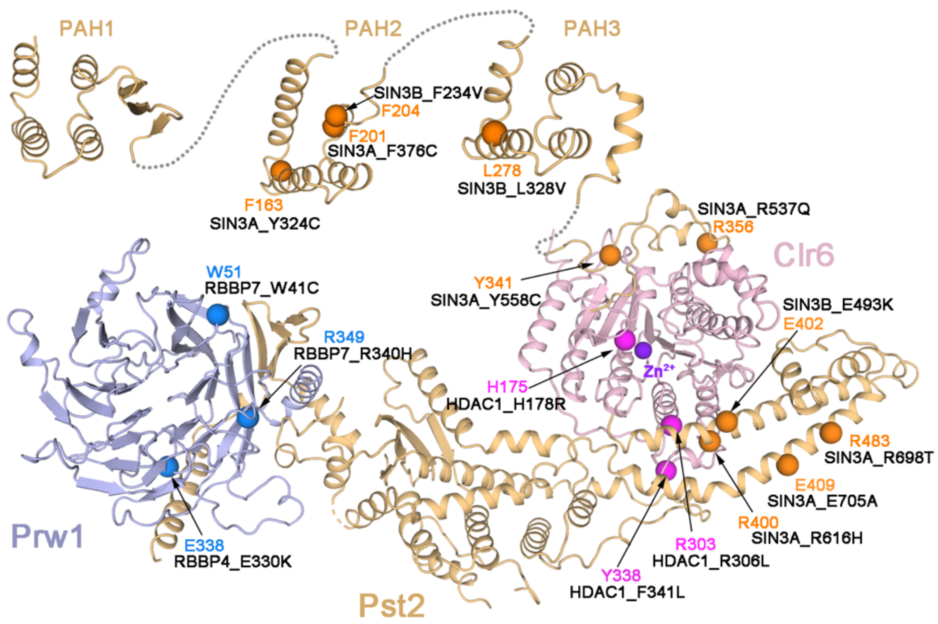

Supplementary Fig. S15 Several invariant residues harboring mutations from patients with cancer, plotted on the structure of Pst2-Clr6-Prw1.

Spheres depict the locations of several invariant residues harboring mutations from patients with cancer that occur at interfaces between the conserved subunits (mainly including Pst2, Clr6 and Prw1). The structure of PAH1/2/3 of Pst2 are separated from the whole complex to show their hydrophobic pockets, which serve as the binding sites for other proteins. In detail, the residues Pst2_F163/F201/F204/L278 locate at the hydrophobic pocket of the SIN3_PAH2/3 domains, which bind to other proteins; the residues Pst2_Y341/R356/R400/E402 and the residues Clr6_R303/Y338 locate at the interface between SIN3_HID and histone deacetylase; the residues Clr6_H175 locate at the deacetylase active center; the residues Pst2_R483/E409 locate at the interface between Sin3 protein and Sds3 or Dep1; the residues Prw1_W51/R349/E338 locate at the interface between Sin3 protein and WD40-containing protein. All these residues are conserved according to the sequence alignment (Supplementary Figs. S9, S12, and S14a).

Supplementary Table S1 Statistics of EM analysis and model validation.

	SIN3L complex	SIN3S complex
**Data collection**		
EM equipment	FEI Titan Krios	FEI Titan Krios
Voltage (kV)	300	300
Detector	K3	K3
Pixel size (Å)	1.087	1.087
Electron dose (e-/Å^2)	50	50
Defocus range (μm)	1.8~2.3	1.8~2.3
**Reconstruction**		
Software	RELION 3.0	cryoSPARC v3
EMDB code	EMD-35093, 35094, 35095	EMD-35092
Number of particles	389,222	777,199
Symmetry	C1	C1
Final masked resolution (Å)	3.2	2.9
Map sharpening B-factor (Å^2)	-109	-128
**Model building**		
Software	Coot 0.8.9/Chimera	Coot 0.8.9/Chimera
Refinement	phenix.real_space_refine	phenix.real_space_refine
PDB code	PDB: 8I03	PDB: 8I02
Protein residues	3,634	2,457
B factors (Å^2)	61.28	39.52
**Validation**		
R.m.s deviations		
Bonds length (Å)	0.010	0.008
Bonds Angle (°)	0.840	0.857
Ramachandran plot statistics (%)		
Favored	92.71	87.41
Outlier	0.06	0,66
Clashscores	15.67	13.37
CaBLAM outliers (%)	2.9	5.7
MolProbity score	3.07	3.00

Supplementary Table S2 Results of mass spectrometry analysis for Pst3-3x-Flag.

Supplementary Table S3 Results of mass spectrometry analysis for Pst2-3x-Flag.
